# BTSR-Net: Boundary–topology guided structural refinement for high-resolution remote sensing image segmentation

**DOI:** 10.1371/journal.pone.0360524

**Published:** 2026-10-16

**Authors:** Ziheng Wang, Wu Wang, Zhe Hu, Yang Li, Kejia Ma

**Affiliations:** 1 School of Electronic and Information Engineering, Changchun University of Science and Technology, Changchun, Jilin, China; 2 North Navigation Control Technology Co., Ltd., No. 2 Kechuang Street, Beijing Economic-Technological Development Area, Beijing, China; 3 Unit of the People’s Liberation Army, Baicheng, Jilin, China; University of Electronic Science and Technology of China, CHINA

## Abstract

Semantic segmentation of high-resolution remote sensing imagery in complex urban scenes often suffers from insufficient modeling of boundary neighborhoods, causing ambiguity near class interfaces and weakening the continuity of elongated structures. To address this issue, we propose a Boundary-Topology-Guided Structural Refinement Network (BTSR-Net). Built upon HRNet-OCR, BTSR-Net introduces a Geometric Boundary–Distance Branch (GBD) with joint boundary–distance supervision to capture spatial transitions around class boundaries, a Multi-scale Topology-Guided Structural Fusion (MTSF) module to enhance connectivity representation of elongated structures, and a dual-stream structural refinement mechanism to perform local residual correction within a boundary-distance-defined refinement narrow-band. Experiments on the ISPRS Potsdam and Vaihingen datasets demonstrate improvements in overall segmentation accuracy, boundary quality, and structural connectivity. On Potsdam, BTSR-Net achieves 84.70% mIoU, 43.17% mBIoU, and 81.63% clDice, exceeding the baseline by 2.91, 5.66, and 3.74 percentage points, respectively. On Vaihingen, it achieves 93.94% mIoU, 73.90% mBIoU, and 95.46% clDice, with corresponding improvements of 2.27, 9.39, and 2.12 percentage points. These results demonstrate the effectiveness of BTSR-Net for structure-sensitive semantic segmentation in high-resolution remote sensing imagery.

## Introduction

High-resolution remote sensing imagery provides fine-grained and information-rich spatial information for applications such as urban modeling [1], land-cover mapping [2,3], and environmental monitoring [4,5], making pixel-level semantic segmentation a fundamental technique for remote sensing image interpretation. Compared with natural-scene imagery, remote sensing images often contain more complex spatial arrangements of objects, including intertwined buildings and roads, transition zones between trees and low vegetation, and small objects such as vehicles embedded in large-scale backgrounds. These characteristics make object contours more irregular, boundaries more fragmented, and transition regions more texturally complex. As a result, segmentation models are prone to boundary drift and local misclassification near class interfaces, as well as structural breaks or adhesion artifacts in elongated targets. Although existing semantic segmentation models provide strong region-level semantic representations, they still lack explicit constraints on contour alignment and structural connectivity. This limitation is particularly evident in elongated structures and complex transition regions, where structural errors tend to accumulate and degrade overall segmentation quality.

To address these challenges, existing studies have improved remote sensing semantic segmentation from several directions. Boundary-aware methods explicitly introduce structural cues to strengthen contour modeling. Representative approaches such as Gated-SCNN [6], SegFix [7], Active Boundary Loss [8], and BASeg [9] improve boundary localization through shape-stream design, direction-field correction, boundary alignment constraints, or boundary-guided context aggregation, thereby reducing error propagation near class interfaces. Local refinement methods, including PointRend [10] and SegRefiner [11], further improve boundary quality by correcting uncertain predictions and repairing local fragmentation in high-resolution masks. However, these methods mainly focus on contour localization or local correction near class interfaces, while the long-range connectivity of elongated and fragmented structures is not explicitly constrained. Meanwhile, Transformer [12]-based architectures provide stronger global semantic representations for dense prediction by strengthening long-range dependency modeling. Representative examples include SegFormer [13], which employs a hierarchical Transformer encoder to produce multi-scale semantic features with high efficiency; Swin Transformer [14], which models cross-region dependencies efficiently by shifted-window attention; and UNetFormer [15], which combines global and local attention within the U-Net [16] framework to balance semantic representation and detail recovery. Nevertheless, stronger contextual representation alone does not directly guarantee accurate boundary alignment or structural connectivity, especially in narrow transition regions and elongated targets.

Beyond boundary modeling and local refinement, preserving structural connectivity has become an important research direction. Methods such as clDice [17], Skeleton Recall Loss [18], and Adaptive Structure-Aware Connectivity-Preserving Loss [19] improve connectivity preservation in elongated or network-like targets through skeleton-based constraints, breakpoint-aware learning, and structure-aware connectivity losses. For remote sensing road segmentation, TopoRF-Net [20] explicitly treats connectivity preservation as a learning objective and introduces topology-related design in the decoding stage to reduce road disconnections. TopoLoRA-SAM [21] further explores topology-preserving supervision under cross-domain adaptation, while Mask2Former [22] provides a flexible structured prediction interface through its unified mask-classification framework. Meanwhile, structural refinement methods such as RefineNet [23], MaskFormer [24], K-Net [25], MaskDiffusion [26], and DifFSS [27] improve boundary detail recovery and local structural correction through multi-path refinement, mask-level representation, iterative updating, and diffusion-based refinement. Although these approaches improve structural continuity or local prediction quality, topology-oriented constraints generally emphasize connectivity preservation, whereas refinement methods mainly focus on correcting local prediction errors. As a result, boundary geometry and structural connectivity are often modeled separately rather than being jointly exploited for targeted refinement. Despite these advances, simultaneously improving boundary adherence and structural connectivity remains difficult in high-resolution remote sensing scenes. This limitation is particularly pronounced in boundary neighborhoods near class interfaces, where boundary ambiguity and structural discontinuity may occur simultaneously and errors are more likely to accumulate.

To address the above issues, we propose the Boundary-Topology-Guided Structural Refinement Network (BTSR-Net) for fine-grained segmentation of high-resolution remote sensing imagery. In this work, topology specifically refers to the preservation of spatial connectivity among same-class regions, particularly elongated and connected structures, rather than to broader topological properties. Built upon HRNet-OCR as the backbone, the proposed network introduces boundary and topology branches while maintaining region-level semantic consistency, and constrains the refinement process to a specifically defined DSR refinement narrow-band for local error correction, thereby reducing structural errors such as boundary drift, disconnections, and adhesion. The main contributions of this work are summarized as follows:

(1) We propose a Geometric Boundary–Distance Branch (GBD) with joint boundary–distance supervision, which jointly models the GBD boundary supervision band and its associated distance-level information, thereby improving contour alignment and discrimination within boundary neighborhoods. Different from conventional boundary-aware approaches that mainly emphasize edge enhancement, GBD jointly models boundary localization and distance-level distributions to characterize spatial transitions within boundary neighborhoods.
(2) We design a Multi-scale Topology-Guided Structural Fusion (MTSF) mechanism that continuously modulates semantic feature alignment with connectivity-oriented topology cues, enhancing the connectivity representation of elongated structures while reducing disconnections and erroneous connections. Unlike topology-aware approaches that mainly impose structural constraints at the output or loss level, MTSF introduces connectivity-oriented topology cues into intermediate multi-scale feature representations and uses them to guide semantic feature fusion.
(3) We develop a localized structural refinement strategy that restricts residual correction to the specifically defined DSR refinement narrow-band, enabling targeted correction around class boundaries without unnecessarily modifying large non-boundary areas. Different from refinement methods that apply updates over the entire prediction, DSR jointly exploits boundary-related and topology-related structural cues within this refinement region.

## Related work

### General semantic segmentation methods

Semantic segmentation of high-resolution imagery requires a balance between semantic discriminability and the preservation of spatial details. Early CNN-based methods, including FCN [28], SegNet [29], and LinkNet [30], established the encoder–decoder paradigm and improved spatial reconstruction through fully convolutional design, pooling-index reuse, and lightweight feature reuse. Subsequent models such as PSPNet [31] and OCRNet [32] enhanced semantic consistency by incorporating multi-scale and object-level contextual modeling. More recently, Transformer-based architectures have extended semantic segmentation toward sequence modeling and unified mask representation. Representative methods such as SETR [33], Mask2Former [22], and RTFormer [34] strengthened long-range dependency modeling, unified multi-task segmentation, and improved the trade-off between efficiency and accuracy, while SegNeXt [35] further improved feature interaction under efficient computation. In high-resolution scenarios such as remote sensing, methods such as SDLED [36] also introduced edge or contour supervision to better preserve structural details. Overall, these methods have substantially improved semantic representation and context modeling. However, their explicit modeling of complex boundaries and structural connectivity remains limited, which has motivated subsequent studies to further investigate the role of boundary and topological information in segmentation.

### Boundary-aware segmentation methods

Boundary-aware segmentation methods aim to improve localization accuracy at class boundaries while preserving regional semantic consistency. In remote sensing scenarios, boundary information has increasingly been introduced into feature learning as an independent structural cue. Representative methods such as BEDSN [37], CSBNet [38], and DSTBA-Net [39] incorporate boundary cues through dual-stream design, class-guided interaction, or boundary-aware feature fusion to enhance contour localization and structural representation. Meanwhile, BEMS-UNetFormer [40] and BCFENet [41] further improve boundary quality by introducing boundary-enhanced attention and edge-prior-based cross-scale feature enhancement. Boundary constraints have also been extended beyond fully supervised settings. BS-GAN [42], Semi-BSU [43], and HR-BMNet [44] demonstrate that boundary-aware learning can improve segmentation quality under semi-supervised, pseudo-label-refined, or multi-resolution learning settings. Overall, boundary information has evolved from an auxiliary supervision signal into an important structural cue in remote sensing semantic segmentation. However, most existing methods still emphasize edge enhancement itself, while continuous geometric modeling of spatial transitions in boundary neighborhoods remains relatively limited.

### Topology-aware segmentation methods

In high-resolution image segmentation, road networks, rivers, and other elongated structures exhibit evident topological properties, making structural connectivity difficult to preserve under pixel-level supervision alone. To address this issue, recent studies have introduced topological constraints into semantic segmentation. Representative methods such as clDice [17], Skeleton Recall Loss [18], and Topology-aware Segmentation Losses [45] improve topological consistency through skeleton-based supervision, breakpoint-aware learning, or topology-preserving loss design. In remote sensing scenarios, topology-aware modeling has also been explored for road extraction and other line-structured targets. BT-RoadNet [46], Topology-Aware Multi-Supervised GAN [47], and TopoRF-Net [20] introduce spatial context modeling, centerline supervision, or topology-preserving constraints to improve connectivity under complex scene conditions. Overall, existing methods have significantly improved structural connectivity through skeleton constraints or topological losses. However, these constraints are mostly imposed at the output layer or through loss functions, and their influence on structural representation in intermediate features remains limited. Therefore, continuously introducing topological cues at the feature level and reinforcing structural consistency in multi-scale representations remains an important research direction.

### Segmentation refinement methods

Segmentation refinement methods aim to correct local prediction errors on top of initial segmentation results, especially residual errors around boundaries and fragmented structural regions. Representative approaches such as K-Net [25], Semantic Diffusion Network [48], MaskDiffusion [26], and SAMRefiner [49] refine masks through iterative updates, diffusion-based denoising, generative correction, or prompt-guided revision, thereby improving boundary quality and structural detail recovery. In remote sensing scenarios, refinement has also been explored for high-resolution labels and feature representations. IUR-Net [50] and GPINet [51] improve refinement quality through multi-stage label updating and feature-level geometric guidance, respectively. Overall, these methods can enhance prediction details at different levels, but most refinement strategies still rely on globally uniform updates over the entire prediction, with limited attention to targeted correction in localized boundary regions. This leaves room for further exploration of structure-guided local refinement.

## Method

### Overall network architecture

To address blurred object boundaries and disrupted topological connectivity in high-resolution remote sensing imagery, we propose a semantic segmentation network termed Boundary-Topology-Guided Structural Refinement Network (BTSR-Net). The network is built upon the multi-resolution representations of HRNet and the object-contextual aggregation of OCR as the main semantic pathway. The Geometric Boundary–Distance Branch (GBD) and Multi-scale Topology-Guided Structural Fusion (MTSF) operate in parallel on the shared multi-scale backbone features to provide complementary boundary-related geometric cues and topology-aware structural representations, respectively. Subsequently, a Dual-Stream Structural Refinement (DSR) is applied at the decoding stage to utilize these structural cues for local correction of semantic predictions within boundary narrow-band regions. While preserving the regional consistency learned by the main segmentation branch, BTSR-Net further enhances geometric awareness through structural guidance and narrow-band refinement. The overall architecture of the proposed network is illustrated in Fig 1.

**Fig 1:**
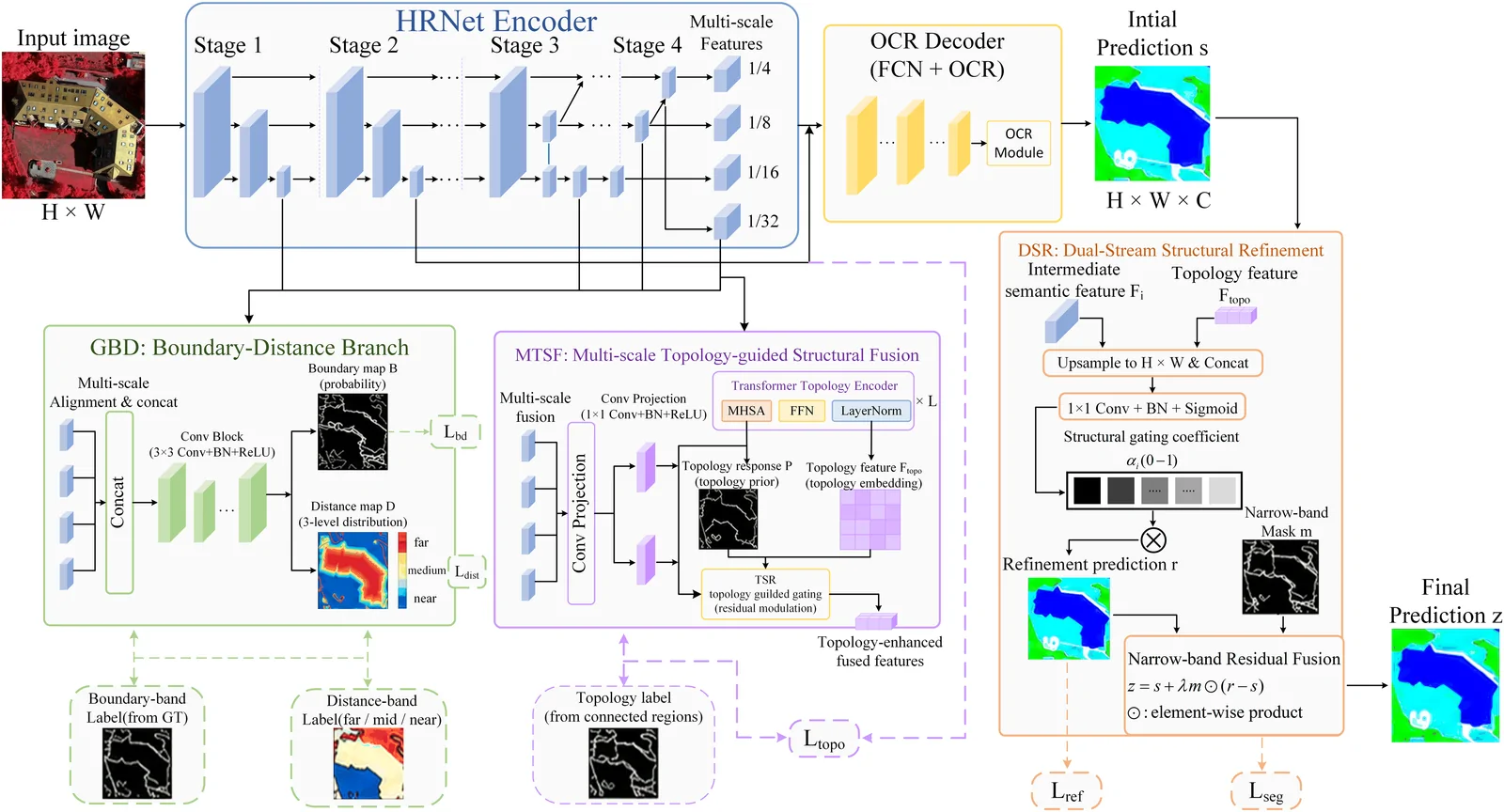
Overall architecture of BTSR-Net.

Along the main pathway, BTSR-Net adopts HRNet as the encoder to preserve high-resolution feature representations and combines it with the OCR decoder head to achieve multi-scale semantic aggregation and initial segmentation prediction. HRNet preserves spatial details and semantic context through parallel multi-resolution branches and continuous information exchange, thereby providing a rich multi-resolution feature foundation for subsequent structural modeling. The OCR module enhances category consistency through object-level region relationship modeling, thereby improving semantic consistency over large spatial regions. On this basis, the network further incorporates GBD, MTSF, and DSR. Specifically, GBD predicts boundary probabilities and distance distributions from multi-scale features to characterize object contours and spatial variations around class boundaries. MTSF adaptively modulates multi-scale features using topological cues to preserve continuous representations of elongated structures and connected regions in feature space. At the decoding stage, DSR integrates semantic predictions with structural cues and performs local correction within the DSR refinement narrow-band. GBD and MTSF operate in parallel on the shared multi-scale backbone features to model complementary boundary-related and topology-related structural information. The resulting structural cues are subsequently utilized by DSR at the downstream refinement stage to perform local correction within the DSR refinement narrow-band. While preserving the regional consistency modeling capability of the main segmentation branch, BTSR-Net further strengthens geometric constraint representation through structural guidance and localized refinement. As a result, the network better preserves object morphology in regions with complex textures, fragmented targets, and elongated structures.

### Geometric boundary–distance branch

In remote sensing imagery, the category boundaries of objects such as roads and buildings are often accompanied by texture mixing and shape transitions in their surrounding neighborhoods. In this work, we use “boundary neighborhood” as a general descriptive term for the spatial vicinity of a class boundary, whereas the “GBD boundary supervision band” specifically refers to the fixed-width region generated from the ground-truth boundary for boundary and distance supervision. If the main segmentation network relies solely on region-level semantic supervision, it is prone to boundary ambiguity and misclassification within these boundary neighborhoods. To introduce explicit boundary- and distance-aware geometric constraints into semantic prediction, we construct a Geometric Boundary–Distance Branch (GBD) in parallel with the main segmentation pathway. By jointly learning boundary probabilities and distance-level distributions, this branch provides continuous spatial cues around class boundaries to support the main segmentation branch. GBD takes the multi-scale features generated by the backbone network as input. First, features from different scales are upsampled to a unified resolution and concatenated to construct the multi-scale fused feature representation:


$$\begin{array}{c} F=Concat\left({\left\{Resize(F_{i})\right\}}_{i=1}^{N}\right) \end{array}$$ (1)

The concatenated features are then projected through a convolutional block to obtain structural features:


$$\begin{array}{c} F_{s}=\phi (F) \end{array}$$ (2)

where ϕ(·) denotes a convolution–normalization–activation operation. Based on this structural feature, GBD predicts the boundary probability map and the distance distribution map in parallel. The boundary sub-branch outputs single-channel boundary logits via a 1×1 convolution, which are then passed through a Sigmoid function to obtain the boundary probability.


$$\begin{array}{c} B=\sigma \left({Conv}_{1\times 1}^{b}(F_{s})\right) \end{array}$$ (3)

This probability indicates how likely a pixel is to belong to the GBD boundary supervision band. The distance sub-branch outputs three-channel distance logits via a 1×1 convolution, which are then normalized by Softmax to obtain the discrete distance distribution:


$$\begin{array}{c} D=Softmax\left({Conv}_{1\times 1}^{d}(F_{s})\right) \end{array}$$ (4)

This distribution describes the relative distance level of each pixel to the nearest boundary (far, medium, or near).

The supervision signals are constructed directly from the semantic labels. First, the initial boundary labels are determined according to category differences within the local neighborhood:


$$\begin{array}{c} B^{gt}(p)=\left\{ \begin{array}{cc} 1, & \exists q\in 𝒩(p),\;Y(q)\neq Y(p)\\ 0, & \text{otherwise} \end{array}\right. \end{array}$$ (5)

where 𝒩(p) denotes the 8-neighborhood of pixel *p*. Subsequently, a training boundary band with a fixed width *w* is generated through morphological dilation:


$$\begin{array}{c} {\tilde{B}}^{gt}=Dilate(B^{gt},w) \end{array}$$ (6)

This defines the GBD boundary supervision band as a fixed-width region around the ground-truth class boundary rather than restricting supervision to single-pixel contours. For distance supervision, the Euclidean distance from each pixel to the nearest boundary is computed with the boundary as reference and then quantized into three distance-level labels (far, medium, near) according to predefined distance thresholds:


$$\begin{array}{c} D^{gt}(p)=Quantize\left(dist(p,{\tilde{B}}^{gt})\right),\;D^{gt}\in \{0,1,2\} \end{array}$$ (7)

Here, Quantize(·) denotes the operation of assigning each pixel to one of three distance levels, namely far, medium, and near, according to distance thresholds, so as to characterize the spatial hierarchy with respect to the GBD boundary supervision band. During training, the boundary prediction is supervised using a binary cross-entropy loss:


$$\begin{array}{c} {ℒ}_{bd}=-\frac{1}{HW}\sum_{p}\left[{\tilde{B}}^{gt}(p)\log B(p)+\left(1-{\tilde{B}}^{gt}(p)\right)\log\left(1-B(p)\right)\right] \end{array}$$ (8)

The distance prediction is supervised using a multi-class cross-entropy loss:


$$\begin{array}{c} {ℒ}_{dist}=-\frac{1}{HW}\sum_{p}\sum_{k}1\left[D^{gt}(p)=k\right]\log D_{k}(p) \end{array}$$ (9)

Accordingly, the training objective of GBD is defined as the sum of the two losses:


$$\begin{array}{c} {ℒ}_{GBD}={ℒ}_{bd}+{ℒ}_{dist} \end{array}$$ (10)

The joint boundary and distance supervision provides complementary geometric information around class boundaries. The structure of GBD and its supervision design are illustrated in Fig 2.

**Fig 2:**
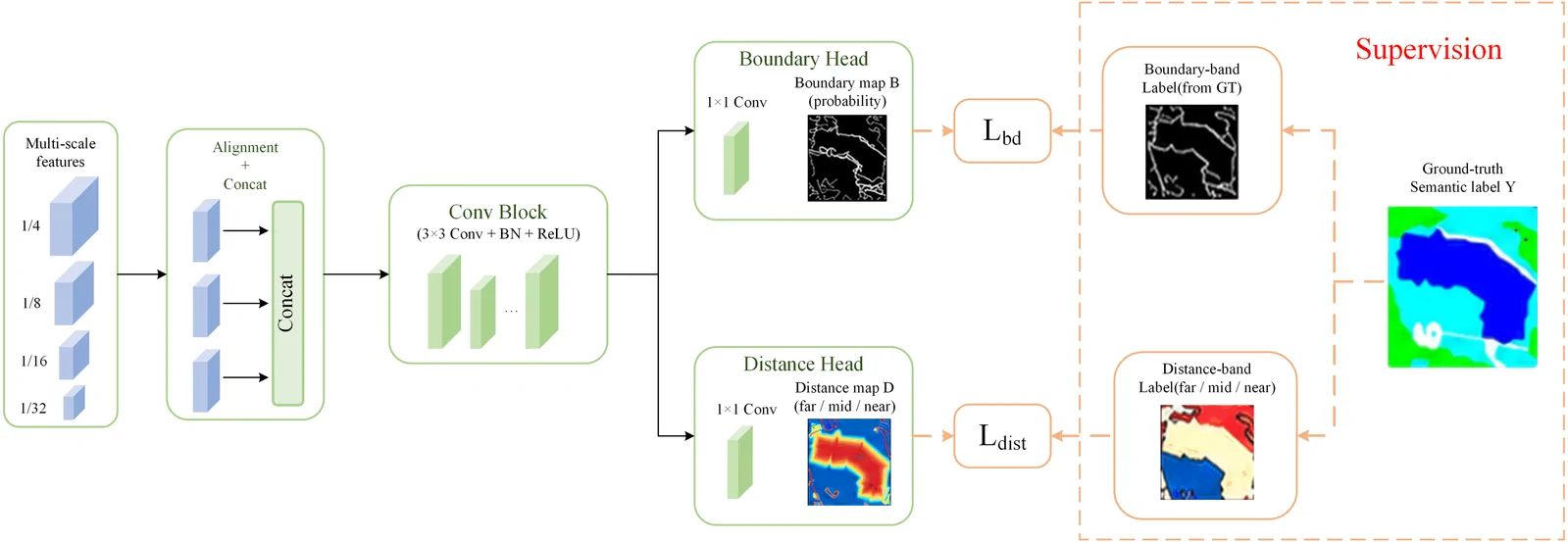
Architecture of the Geometric Boundary–Distance Branch (GBD) and its geometric supervision design.

### Multi-scale topology-guided structural fusion

Roads, building boundaries, and other elongated targets in remote sensing scenes usually exhibit evident connected structures, but under the influence of complex texture interference and scale variation, semantic features are prone to local disconnections or structural adhesion, thereby undermining structural continuity. To enhance the segmentation network’s ability to model connectivity explicitly, we introduce Multi-scale Topology-Guided Structural Fusion (MTSF) on top of the backbone features. Accordingly, the terms topology response, topology feature, and topology cue used below all denote connectivity-oriented representations at different stages of MTSF. MTSF learns connectivity-aware topology representations from multi-scale features and uses them to modulate semantic features along the multi-scale pathways. Within MTSF, a Topology-guided Structural Refinement (TSR) unit transforms the learned topology features into gating responses for semantic feature recalibration. The overall architecture and information flow of MTSF are illustrated in Fig 3.

**Fig 3:**
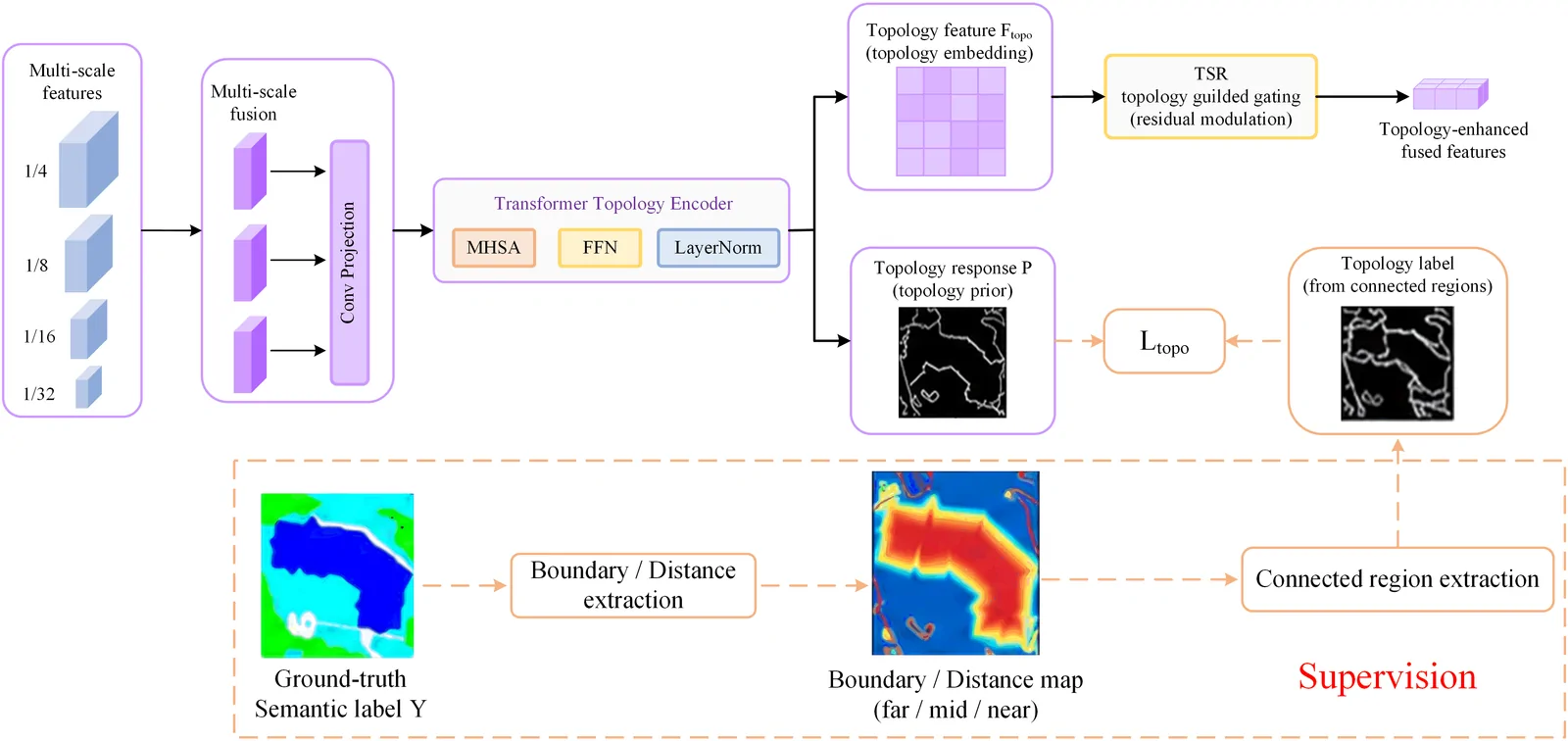
Architecture of the Multi-scale Topology-Guided Structural Fusion (MTSF) module and its topology-guided fusion mechanism.

As shown in Fig 3, MTSF takes the multi-scale features produced by the backbone network as input. Features from different scales are first aligned in resolution and concatenated to obtain the fused feature representation:


$$\begin{array}{c} F_{ms}=Concat\left({\left\{Resize(F_{j})\right\}}_{j=1}^{N}\right) \end{array}$$ (11)

The fused representation is then projected by a convolutional layer to obtain an intermediate feature for topology modeling:


$$\begin{array}{c} F_{t}=\psi (F_{ms}) \end{array}$$ (12)

where ψ(·) denotes a convolution–normalization–activation operation.

To explicitly model structural connectivity, MTSF introduces a topology encoder on $F_{t}$, implemented as a Transformer encoder, which models spatial structural correlations through multi-head self-attention and produces the topology feature:


$$\begin{array}{c} T=Transformer(F_{t}) \end{array}$$ (13)

Based on this topology representation, the module outputs the topology logits and the topology feature map in parallel:


$$\begin{array}{c} P=\alpha (W_{p}*T) \end{array}$$ (14)

$$\begin{array}{c} F_{topo}=\gamma (T) \end{array}$$ (15)

Here, $W_{p}$ denotes a 1×1 convolution, $P\in {\mathbb{R}}^{B\times H\times W}$ represents the pixel-wise connectivity response used for topology supervision, and γ(·) denotes a convolutional mapping.

The topology supervision is derived from the semantic labels. Given the semantic label map *Y*, the topology label of a pixel is set to 1 if it lies inside a connected region of the same class, and 0 otherwise, i.e.,


$$\begin{array}{c} P^{gt}(p)=\left\{ \begin{array}{cc} 1, & p\in C(Y)\\ 0, & \text{otherwise} \end{array}\right. \end{array}$$ (16)

where *C*(*Y*) denotes the set of semantically connected regions. The topology branch is trained using a binary cross-entropy loss:


$$\begin{array}{c} {ℒ}_{topo}=-\frac{1}{HW}\sum_{p}\left[P^{gt}(p)\log P(p)+\left(1-P^{gt}(p)\right)\log\left(1-P(p)\right)\right] \end{array}$$ (17)

During feature fusion, MTSF employs a Topology-guided Structural Refinement (TSR) unit to inject the learned topology information into the semantic features. TSR is an internal feature-recalibration component of MTSF rather than an independent structural branch. Specifically, given the topology feature $F_{topo}$, TSR first projects it through a 1×1 convolution and then applies a Sigmoid function to generate a bounded topology-guided gating response *G*:


$$\begin{array}{c} G=TSR(F_{topo})=\sigma \left({Conv}_{1\times 1}(F_{topo})\right) \end{array}$$ (18)

where σ(·) denotes the Sigmoid function. Let $F_{s}$ denote the semantic-path feature. The generated gating response is then applied to $F_{s}$ through residual multiplicative modulation:


$$\begin{array}{c} F_{fuse}=F_{s}⊙(1+G) \end{array}$$ (19)

where ⊙ denotes element-wise multiplication. In this formulation, TSR serves as the topology-guided feature modulation unit within MTSF: $F_{topo}$ provides the connectivity-oriented topology cues, while the residual term (1 + *G*) adaptively recalibrates the semantic response without directly replacing the original semantic representation.

### Dual-stream structural refinement

The main segmentation branch provides coherent regional semantics, but in boundary neighborhoods, such as road edges, building contours, and the interfaces between fragmented objects and complex textures, it remains prone to errors including boundary over-expansion or shrinkage, as well as local adhesion and disconnections. Such errors are typically concentrated around class boundaries where semantic confidence changes rapidly, and a model trained with region-level supervision alone often lacks sufficient structural guidance in these areas. To address this issue, we introduce Dual-Stream Structural Refinement (DSR) as a downstream refinement module following the parallel structural modeling of GBD and MTSF. DSR integrates topology-aware features with the semantic representations of the main pathway and restricts local residual correction to a specifically defined DSR refinement narrow-band, thereby avoiding unnecessary modifications to regions away from class boundaries. The architecture of the Dual-Stream Structural Refinement branch is illustrated in Fig 4.

**Fig 4:**
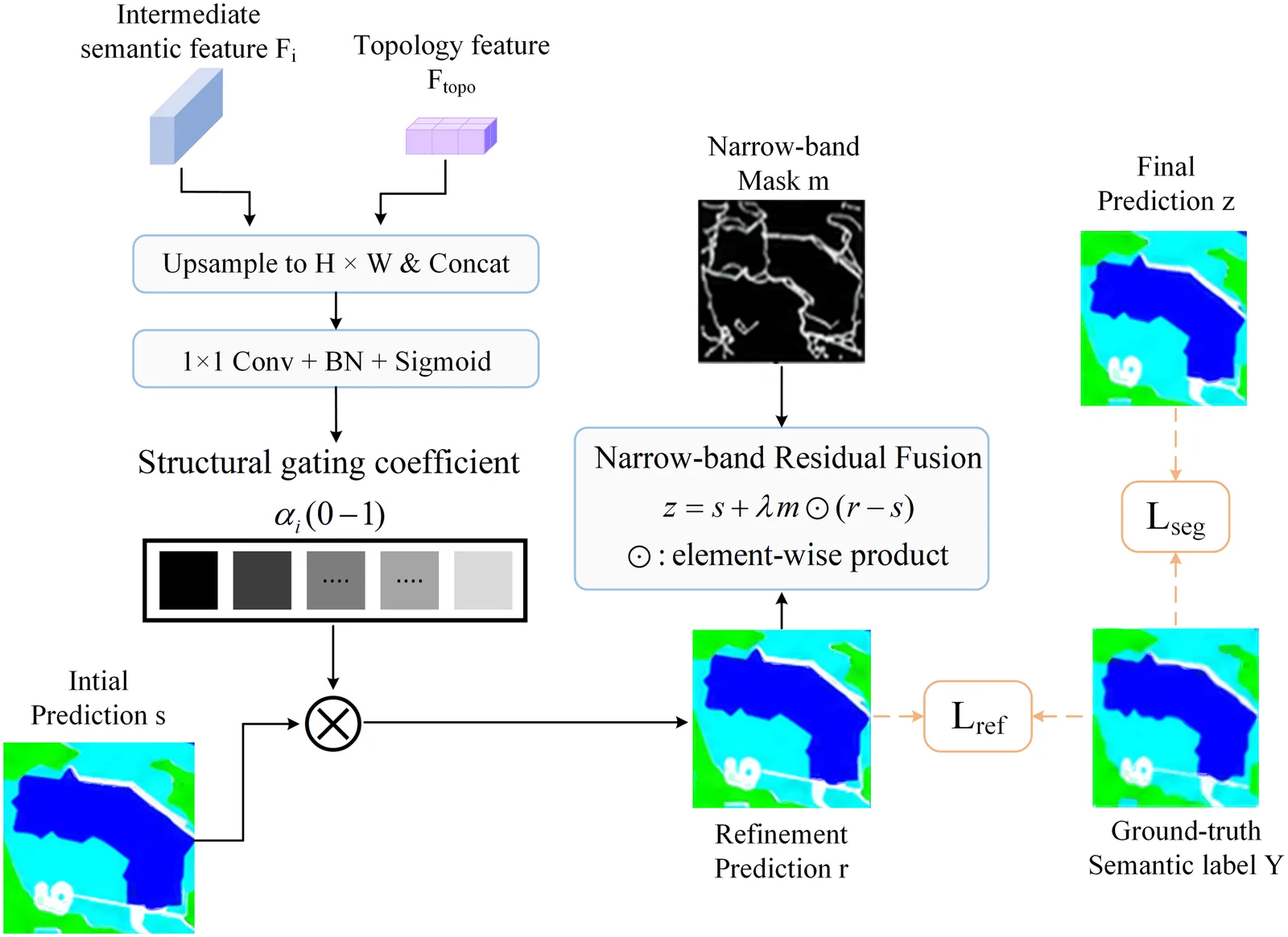
Architecture of the Dual-Stream Structural Refinement (DSR) module.

As shown in Fig 4, the core of DSR is a residual refinement pathway driven by structural information. Let $F_{i}\in {\mathbb{R}}^{B\times C\times H\times W}$ denote the intermediate-to-high-level semantic features from the main segmentation pathway, and $F_{topo}\in {\mathbb{R}}^{B\times C\times h\times w}$ denote the topological features produced by Multi-scale Topology-Guided Structural Fusion (MTSF). The topological features are upsampled to the same resolution as $F_{i}$ and concatenated with it:


$$\begin{array}{c} {\hat{F}}_{topo}=Up(F_{topo}) \end{array}$$ (20)

$$\begin{array}{c} U_{i}=Concat(F_{i},{\hat{F}}_{topo}) \end{array}$$ (21)

The concatenated feature is then passed through a 1×1 convolution and normalization to generate the gating coefficient, corresponding to the Sigmoid output in Fig 4:


$$\begin{array}{c} \alpha_{i}=\sigma \left(BN\left({Conv}_{1\times 1}(U_{i})\right)\right) \end{array}$$ (22)

$$\begin{array}{c} \alpha_{i}\in [0,1{]}^{B\times H\times W} \end{array}$$ (23)

The gating coefficient enhances the original semantic features in a multiplicative residual manner, yielding the refined feature representation:


$$\begin{array}{c} {\tilde{F}}_{i}=F_{i}⊙(1+\beta \cdot \alpha_{i}) \end{array}$$ (24)

Here, ⊙ denotes element-wise multiplication, and β is a scaling factor used to control the modulation strength of the structural gating on the semantic features. The coefficient $\alpha_{i}$ serves as a spatial modulation map for recalibrating the semantic feature $F_{i}$.

Based on ${\tilde{F}}_{i}$, the refinement branch further generates a refinement prediction *r*. To distinguish the refinement region used by DSR from the GBD boundary supervision band ${\tilde{B}}^{gt}$, we explicitly define the DSR refinement narrow-band according to the boundary-distance levels. During training, the ground-truth DSR refinement mask is constructed from the distance label $D^{gt}$ as


$$\begin{array}{c} m^{gt}(p)=1\left[D^{gt}(p)\in \{1,2\}\wedge Y(p)\neq ignore\right], \end{array}$$ (25)

where distance level 0 denotes the far region, while levels 1 and 2 denote the medium and near-boundary regions, respectively. Therefore, only the medium and near-boundary regions participate in the local refinement supervision, whereas far regions away from class boundaries are excluded.

During inference, the predicted distance level is first obtained from the GBD distance distribution as


$$\begin{array}{c} \hat{D}(p)=\text{arg}\max_{k\in \{0,1,2\}}D_{k}(p), \end{array}$$ (26)

and the corresponding DSR refinement narrow-band mask is defined as


$$\begin{array}{c} m(p)=1\left[\hat{D}(p)\in \{1,2\}\right]. \end{array}$$ (27)

The refinement prediction *r* is then combined with the backbone semantic prediction *s* only within the DSR refinement narrow-band through residual fusion:


$$\begin{array}{c} z=s+\lambda m⊙(r-s) \end{array}$$ (28)

where *m* denotes the DSR refinement narrow-band mask and λ is the fusion weight used to control the strength of residual correction. It should be noted that the DSR refinement narrow-band is different from the GBD boundary supervision band ${\tilde{B}}^{gt}$. The latter is a fixed-width region generated by dilating the ground-truth class boundary and is used for boundary supervision in GBD, whereas the former is determined by the medium and near-boundary distance levels and is used specifically to restrict the spatial range of DSR refinement.

### Loss function

To jointly constrain regional semantics, boundary localization, and structural connectivity, BTSR-Net is trained with supervision from both the main segmentation branch and the structural branches. The overall loss consists of the semantic segmentation loss, the GBD geometric supervision loss, the MTSF topology constraint loss, the DSR refinement loss, and an additional boundary–topology consistency loss that coordinates the boundary-distance information learned by GBD with the topology response learned by MTSF.

The main segmentation branch supervises the final semantic prediction *z*, and the semantic label map is denoted by *Y*. Its loss is defined as:


$$\begin{array}{c} {ℒ}_{seg}=CE(z,Y) \end{array}$$ (29)

Here, CE(·) denotes the pixel-wise cross-entropy loss, which is used to ensure regional semantic consistency and class discriminability.

GBD constrains boundary geometry through supervision of the GBD boundary supervision band and the associated distance levels, and its loss is written as:


$$\begin{array}{c} {ℒ}_{GBD}={ℒ}_{bd}+{ℒ}_{dist} \end{array}$$ (30)

Here, ${ℒ}_{bd}$ is the binary cross-entropy loss for boundary prediction, which constrains class boundary locations, and ${ℒ}_{dist}$ is the cross-entropy loss for distance distribution, which constrains the distance levels with respect to the GBD boundary supervision band and thereby improves discrimination between distance levels around class boundaries.

For MTSF, connectivity structures are explicitly modeled through connectivity-oriented topology supervision, and the corresponding loss is given by:


$$\begin{array}{c} {ℒ}_{topo}=-\frac{1}{HW}\sum_{p}\left[P^{gt}(p)\log P(p)+\left(1-P^{gt}(p)\right)\log\left(1-P(p)\right)\right] \end{array}$$ (31)

Here, *P* denotes the predicted connectivity response, and $P^{gt}$ denotes the corresponding topology supervision target derived from the semantic labels as defined above. This term provides full-map connectivity-oriented supervision for MTSF. The boundary–topology consistency constraint introduced below is different from ${ℒ}_{topo}$, as it uses the boundary-distance partition to impose an additional regularization on a specific subset of pixels.

In addition to the topology supervision, a boundary–topology consistency constraint is introduced to coordinate the boundary-distance supervision associated with GBD with the connectivity response of MTSF. Specifically, the boundary-distance labels used for GBD supervision divide pixels into far, intermediate, and near-boundary regions. Pixels in the far region correspond to interior areas away from class boundaries. We therefore encourage the topology branch to maintain high connectivity responses in these regions. The set of valid far-region pixels is defined as


$$\begin{array}{c} \Omega_{far}=\left\{p|D^{gt}(p)=0,\;Y(p)\neq ignore\right\}, \end{array}$$ (32)

where $D^{gt}$ denotes the discrete boundary-distance label used for GBD supervision, with 0 representing the far region. The boundary–topology consistency loss is defined as


$$\begin{array}{c} {ℒ}_{BT}=-\frac{1}{\left|\Omega_{far}\right|}\sum_{p\in \Omega_{far}}\log\sigma \left(P_{logit}(p)\right), \end{array}$$ (33)

where $P_{logit}$ denotes the topology logits predicted by MTSF and σ(·) denotes the Sigmoid function. The boundary–topology consistency loss therefore uses the boundary-distance partition associated with GBD to regularize the connectivity response of MTSF in far regions away from class boundaries. Unlike ${ℒ}_{topo}$, which provides topology supervision over all valid pixels, ${ℒ}_{BT}$ acts only on the far-region subset $\Omega_{far}$ and serves as an auxiliary consistency regularization term.

In the refinement stage, DSR outputs a refinement prediction *r*, and its supervision is restricted to the ground-truth DSR refinement narrow-band $\Omega_{NB}^{gt}$. The refinement loss is defined as:


$$\begin{array}{c} {ℒ}_{ref}=\frac{1}{\left|\Omega_{NB}^{gt}\right|}\sum_{p\in \Omega_{NB}^{gt}}CE(r(p),Y(p)). \end{array}$$ (34)

This term trains the refinement branch only on the medium- and near-boundary regions, preventing large far regions away from class boundaries from dominating the local refinement objective.

The final training objective is given by:


$$\begin{array}{c} ℒ={ℒ}_{seg}+\eta {ℒ}_{GBD}+\mu {ℒ}_{topo}+\nu {ℒ}_{ref}+w_{BT}{ℒ}_{BT} \end{array}$$ (35)

Here, η, μ, and ν denote the weighting coefficients of the GBD geometric supervision loss, MTSF topology loss, and DSR refinement loss, respectively. The coefficient $w_{BT}$ is applied specifically to the boundary–topology consistency term ${ℒ}_{BT}$ and controls the strength of this auxiliary regularization during training. Unless otherwise specified, $w_{BT}$ is set to 0.02 for the full model. The sensitivity of $w_{BT}$ is further evaluated in the ablation experiments.

## Experiments

### Datasets and preprocessing

To comprehensively evaluate the segmentation performance of the proposed BTSR-Net, two widely used high-resolution aerial image datasets provided by the ISPRS (International Society for Photogrammetry and Remote Sensing), namely Potsdam and Vaihingen, are used for experiments. Both datasets provide pixel-level annotations with consistent category definitions, consisting of impervious surface (white), building (blue), low vegetation (cyan), tree (green), car (yellow), and clutter (red). Due to large-scale object variation and complex boundary structures in urban scenes, segmentation models are prone to boundary drift, fragmentation, and adhesion errors, particularly near building contours, road boundaries, and small objects such as vehicles. Therefore, comparison experiments and ablation studies are conducted on these two datasets to evaluate the effectiveness of the proposed method in terms of segmentation accuracy, boundary accuracy, and structural connectivity.

### ISPRS potsdam dataset

The Potsdam dataset, released by ISPRS, contains 38 orthorectified aerial images (TOP), each with a resolution of 6000 × 6000 pixels and a spatial resolution of 5 cm/pixel. In this dataset, buildings exhibit regular structures with clear boundaries, impervious surfaces and roads are widely distributed, and vehicles frequently appear as small objects. Because of the large number of long edges, sharp corners, and slender structures, segmentation models tend to produce jagged boundaries, contour discontinuities, and misclassifications near class transitions. Therefore, the Potsdam dataset is suitable for evaluating the ability of a model to preserve boundary accuracy and structural connectivity.

### ISPRS vaihingen dataset

The Vaihingen dataset, released by ISPRS, contains 33 high-resolution aerial images with a size of approximately 2000 × 2500 pixels and a spatial resolution of 9 cm/pixel. Compared with Potsdam, the Vaihingen dataset exhibits greater variations in scene texture, roof materials, and shadow distribution, and vegetation regions are more susceptible to texture interference and class confusion, while building boundaries are more likely to become blurred or partially missing due to shadows. This dataset is suitable for evaluating the generalization capability of segmentation models under different scene distributions and imaging conditions, and is particularly useful for examining the robustness of boundary accuracy and structural connectivity under complex backgrounds and illumination variations.

### Data preprocessing and dataset split

Remote sensing images usually have very large spatial sizes, and directly inputting the original images into deep neural networks may lead to excessive memory consumption and reduced training efficiency. To address this issue, a sliding-window cropping strategy is adopted to divide large images into smaller patches suitable for network training. During cropping, overlapping regions are introduced to preserve contextual continuity and reduce prediction inconsistency near patch boundaries.

For the Potsdam dataset, the original data consist of 38 large images with a resolution of 6000 × 6000 pixels. An overlap ratio of 0.7 is adopted, and the images are cropped into patches of size 512 × 512, resulting in 7448 image samples. The cropped data are organized in the VOC-style directory format and divided into 5958 training images and 1490 validation images for subsequent model training and evaluation.

For the Vaihingen dataset, the original data contain 33 images. The same cropping strategy and parameter settings as those used for Potsdam are applied, producing 4693 image patches, among which 3754 are used for training and 939 for validation. This preprocessing pipeline ensures consistent cropping size, overlap settings, and train–validation splits for both datasets, providing a unified basis for fair comparison and ablation analysis.

### Experimental settings and evaluation metrics

This section describes the implementation details and evaluation protocols used in the experiments. All comparative and ablation experiments are conducted under the same framework and training configuration. Only the dataset paths and split files are changed between Potsdam and Vaihingen to ensure consistent settings for fair comparison.

### Experimental settings

All experiments are implemented using MMSegmentation, with a fixed input size of 512 × 512 and six semantic classes. Data preprocessing uses fixed mean and variance for normalization, and the ignore label is set to 255. The network uses HRNet-W48 as the backbone with pretrained weights, and uses OCRHead as the decoder and FCNHead as the auxiliary head. Both the main branch and the auxiliary branch are supervised using cross-entropy loss, and the auxiliary loss weight is set to 0.4. The structural modeling component includes a Geometric boundary–distance branch, a topology branch, and a structural refinement branch. The boundary branch is supervised using binary cross-entropy, the distance levels are supervised using cross-entropy, and the topology branch provides structural guidance through multi-scale gated fusion. The structural refinement branch is supervised only within the ground-truth DSR refinement narrow-band during training, while residual fusion is restricted to the predicted DSR refinement narrow-band during inference to avoid unnecessary modification of regions away from class boundaries. Potsdam and Vaihingen use the same network architecture and hyperparameter settings, with only the dataset directories and split files changed.

The optimizer is SGD with an initial learning rate of 0.01, momentum of 0.9, and weight decay of $5\times 10^{-4}$. The learning rate follows the Poly decay policy with power = 0.9 and $\eta_{\min}=1\times 10^{-4}$. Training is iteration-based with a maximum of 80,000 iterations, and validation is performed every 4,000 iterations. The best model is selected according to mIoU. The batch size is 4 for training and 2 for validation. Sliding-window inference is used during testing, with a window size of 512×512 and a stride of 341×341.

All experiments are conducted on Windows 11 using CUDA 12.1 and PyTorch 2.1 with Python 3.10.19. The hardware configuration is shown in Table 1, with an NVIDIA GeForce RTX 4070 Ti SUPER GPU and an Intel i5-13490F CPU.

**Table 1:** Experimental Environment and Operating Platform.

Environment	Type
Operating System	Windows 11
Framework	CUDA 12.1 and PyTorch 2.1
Language	Python 3.10.19
CPU	13th Gen Intel(R) i5-13490F
GPU	NVIDIA GeForce RTX 4070 Ti SUPER

### Evaluation metrics

The following commonly used semantic segmentation metrics are adopted to quantitatively evaluate the performance of the proposed method:

Mean Intersection over Union (mIoU): Intersection over Union (IoU) is a widely used metric for measuring the overlap between the predicted results and the ground truth for each class, defined as follows:


$$\begin{array}{c} IoU_{i}=\frac{TP_{i}}{TP_{i}+FP_{i}+FN_{i}} \end{array}$$ (36)

The mean IoU (mIoU) is obtained by averaging IoU over all classes:


$$\begin{array}{c} mIoU=\frac{1}{C}\sum_{i=1}^{C}IoU_{i} \end{array}$$ (37)

where TP, FP, and FN denote true positives, false positives, and false negatives, respectively. TP represents the number of pixels correctly predicted as the target class, FP denotes pixels incorrectly predicted as the target class, FN denotes pixels belonging to the target class but predicted otherwise, and *C* denotes the total number of classes. This metric measures the overlap between predicted regions and ground-truth regions and is one of the most representative indicators for segmentation accuracy.

Mean F1 score (mF1): The F1 score evaluates the balance between precision and recall of the model, defined as follows:


$$\begin{array}{c} {Precision}_{i}=\frac{TP_{i}}{TP_{i}+FP_{i}} \end{array}$$ (38)

$$\begin{array}{c} {Recall}_{i}=\frac{TP_{i}}{TP_{i}+FN_{i}} \end{array}$$ (39)

$$\begin{array}{c} F1_{i}=\frac{2\cdot {Precision}_{i}\cdot {Recall}_{i}}{{Precision}_{i}+{Recall}_{i}}=\frac{2\cdot TP_{i}}{2\cdot TP_{i}+FP_{i}+FN_{i}} \end{array}$$ (40)

$$\begin{array}{c} mF1=\frac{1}{C}\sum_{i=1}^{C}F1_{i} \end{array}$$ (41)

The mF1 summarizes the class-wise balance between precision and recall and serves as a complementary region-based metric to IoU.

Overall Accuracy (OA): OA measures the proportion of correctly predicted pixels among all predictions, defined as follows:


$$\begin{array}{c} OA=\frac{\sum_{i=1}^{C}TP_{i}}{\sum_{i=1}^{C}(TP_{i}+FP_{i}+FN_{i}-TP_{i})}=\frac{\sum_{i=1}^{C}TP_{i}}{\sum_{i=1}^{C}N_{i}} \end{array}$$ (42)

OA is a global metric reflecting overall classification accuracy over all pixels and is useful for evaluating the overall consistency of segmentation results.

To better evaluate boundary quality, two boundary-aware metrics, mBIoU and mBFScore, are adopted. For each class, the binary mask $M_{i}$ is first converted into a boundary set $B(M_{i})$. Here, B(·) denotes the boundary extraction operator, which can be implemented using morphological gradients or contour extraction. Considering that slight misalignment may exist between pixel-level annotations and predictions, a tolerance bandwidth *r* is introduced. A dilation operator ⊕ is used to construct the tolerance region $B(M_{i})⊕D(r)$, where *D*(*r*) is a structuring element with radius *r*. For class *i*, the boundary-level true positives, false positives, and false negatives are defined as follows:


$$\begin{array}{c} TP_{i}^{b}=\left|B(P_{i})\cap (B(G_{i})⊕D(r))\right| \end{array}$$ (43)

$$\begin{array}{c} FP_{i}^{b}=\left|B(P_{i})⧵(B(G_{i})⊕D(r))\right| \end{array}$$ (44)

$$\begin{array}{c} FN_{i}^{b}=\left|B(G_{i})⧵(B(P_{i})⊕D(r))\right| \end{array}$$ (45)

Based on these definitions, boundary IoU and boundary F1 are computed as follows:


$$\begin{array}{c} BIoU_{i}=\frac{TP_{i}^{b}}{TP_{i}^{b}+FP_{i}^{b}+FN_{i}^{b}} \end{array}$$ (46)

$$\begin{array}{c} BFScore_{i}=\frac{2\cdot TP_{i}^{b}}{2\cdot TP_{i}^{b}+FP_{i}^{b}+FN_{i}^{b}} \end{array}$$ (47)

The average across all categories is:


$$\begin{array}{c} mBIoU=\frac{1}{C}\sum_{i=1}^{C}BIoU_{i} \end{array}$$ (48)

$$\begin{array}{c} mBFScore=\frac{1}{C}\sum_{i=1}^{C}BFScore_{i} \end{array}$$ (49)

These metrics focus on boundary accuracy and shape consistency, and are more sensitive to structural details such as road edges and building boundaries. To evaluate structural connectivity and topological consistency, the clDice (mean) metric is adopted. For each class *i*, the predicted binary mask $P_{i}$ and the ground-truth binary mask $G_{i}$ are skeletonized, and the skeletonization operator is denoted as S(·). The topological precision and topological recall are defined as:


$$\begin{array}{c} tPrec_{i}=\frac{\left|S(P_{i})\cap G_{i}\right|}{\left|S(P_{i})\right|} \end{array}$$ (50)

$$\begin{array}{c} tSens_{i}=\frac{\left|S(G_{i})\cap P_{i}\right|}{\left|S(G_{i})\right|} \end{array}$$ (51)

The average across all categories is:


$$\begin{array}{c} clDice(mean)=\frac{1}{C}\sum_{i=1}^{C}clDice_{i} \end{array}$$ (52)

This metric is more sensitive to structural breaks, holes, and incorrect connections in thin structures, complementing region-based metrics that are less sensitive to topological errors.

Number of parameters (Params): This metric measures the total number of trainable parameters in the model and is an important indicator of model complexity and memory cost. Each parameter corresponds to a learnable weight updated during training; larger parameter size generally indicates stronger representation ability but higher computational cost. The number of parameters is calculated as follows:


$$\begin{array}{c} Params=\sum_{i=1}^{L}\prod_{j}d_{ij} \end{array}$$ (53)

Here, *L* is the number of learnable tensors, and $d_{ij}$ denotes the size of each dimension of the *i*-th tensor. GFLOPs represent the number of floating-point operations in one forward inference, usually counted as multiply–accumulate operations and normalized by 10^9^. Taking convolution as an example, if the output feature size is H×W, the number of input channels is $C_{in}$, the number of output channels is $C_{out}$, the convolution kernel size is K×K, and the number of groups is *g*, then the approximate computational cost is:


$$\begin{array}{c} {FLOPs}_{conv}\approx 2\cdot H\cdot W\cdot C_{out}\cdot \frac{C_{in}}{g}\cdot K^{2} \end{array}$$ (54)

$$\begin{array}{c} GFLOPs=\frac{\sum FLOPs}{10^{9}} \end{array}$$ (55)

Parameters and GFLOPs reflect the model’s computational overhead from the perspectives of memory usage and computation, respectively, facilitating a comprehensive comparison between accuracy and efficiency. All the above metrics are computed at the pixel level and averaged over all classes to obtain a comprehensive and fair evaluation.

### Comparison with existing methods

To comprehensively evaluate the segmentation performance of BTSR-Net on the ISPRS Potsdam and Vaihingen datasets, several representative methods from different categories are selected for comparison. U-Net [16], PSPNet [31] and DeepLabV3+ [52] are selected as representative convolutional models, representing traditional encoder–decoder architectures and multi-scale context modeling methods in remote sensing segmentation; CCNet [53] and OCRNet [32] adopt context aggregation strategies similar to the semantic branch used in this work, allowing evaluation of the impact of semantic aggregation capability; PointRend [10] focuses on point-based prediction and local refinement, mainly targeting boundary correction; SegFormer [13], Swin-UperNet [14], Segmenter [54] and DPT [55] represent Transformer-based or hybrid architectures that emphasize global dependency modeling and strong feature representation. These methods are used to evaluate the overall modeling capability of BTSR-Net beyond structural constraints. UNetFormer [15], LoG-CAN [56] and BevaNet [57] focus on structural enhancement and feature interaction design for remote sensing segmentation, which are related to the boundary modeling, topology modeling, and localized DSR refinement design of the proposed method. These methods include both general-purpose segmentation models and approaches specialized in boundary refinement, structural modeling, and global context learning, allowing a more objective evaluation of the performance of BTSR-Net under consistent experimental settings.

### Comparison on the potsdam dataset

Table 2 reports the comparison results of different methods on the ISPRS Potsdam dataset. BTSR-Net achieves the best performance in the overall metrics, including mIoU, mF1, and OA. Specifically, BTSR-Net achieves 84.70% mIoU, 91.57% mF1, and 92.97% OA, improving the baseline by 2.91%, 1.84%, and 1.41%, respectively. From the class-wise perspective, the advantage of BTSR-Net is more evident on categories with complex structures and irregular boundaries. For example, the IoU of the Clutter class reaches 74.93%, which is significantly higher than the baseline value of 67.32%. This category usually contains scattered small objects and complex textures, which makes segmentation more challenging. Segmentation models tend to produce fragmented predictions and irregular boundaries in such regions. Improvements are also observed in the Low-veg and Tree categories. The IoU reaches 82.38% for Low-veg and 79.37% for Tree. These two categories often appear in transition regions near roads and buildings, where predictions are prone to shrinkage or boundary noise. Boundary-specific and connectivity-related improvements are supported separately by the structural metrics, where BTSR-Net achieves 43.17% mBIoU and 81.63% clDice (mean), compared with 37.51% and 77.89% for the baseline, respectively. The corresponding boundary and structural differences are further illustrated by the qualitative results in Fig 5.

**Table 2:** Comparison Results for the ISPRS Potsdam dataset.

Methods	Imp-surf	Building	Low-veg	Tree	Car	Clutter	mIoU	mF1	OA	mBIoU	clDice (mean)
U-Net [16]	79.33	87.98	71.09	70.33	79.89	35.93	70.76	81.58	86.19	28.31	60.93
PSPNet [31]	86.97	94.22	78.13	76.63	84.48	65.76	81.03	89.25	91.13	35.38	76.35
DeepLabV3+ [52]	86.39	93.82	77.08	75.86	83.88	63.73	80.13	88.65	90.66	34.62	75.44
SegFormer [13]	81.41	89.37	71.00	65.54	77.42	51.51	72.71	83.61	86.71	29.24	66.60
Swin-UperNet [14]	88.13	94.92	79.67	77.42	85.58	68.26	82.26	90.02	91.80	39.11	78.59
Segmenter [54]	84.67	92.70	75.85	73.13	77.79	60.63	77.46	86.94	89.63	30.20	73.97
DPT [55]	78.48	87.08	67.77	60.57	73.70	49.47	69.51	81.39	84.57	25.85	65.55
PointRend [10]	97.26	94.26	78.77	76.35	84.89	64.73	81.04	89.23	91.26	38.11	76.96
CCNet [53]	88.28	94.98	80.17	78.04	85.70	69.98	82.86	90.42	92.04	38.85	78.83
UNetFormer [15]	86.11	93.87	77.40	75.35	83.74	63.79	80.04	88.60	90.62	35.51	75.49
LoG-CAN [56]	86.94	94.41	78.32	76.16	84.78	64.11	80.79	89.06	91.09	37.48	77.73
BevaNet [57]	82.78	90.82	73.14	69.63	81.00	51.63	74.84	84.99	87.96	31.81	71.96
SeaFormer [58]	69.69	78.38	58.22	44.07	59.68	29.64	56.61	70.88	76.84	15.22	51.48
EfficientViT [59]	79.31	87.93	67.57	59.91	74.02	44.91	68.94	80.78	84.64	24.66	64.56
Baseline	87.60	94.70	79.22	76.86	85.04	67.32	81.79	89.73	91.56	37.51	77.89
**Ours**	**89.72**	**95.58**	**82.38**	**79.37**	**86.24**	**74.93**	**84.70**	**91.57**	**92.97**	**43.17**	**81.63**

For brevity, Imp-surf and Low-veg denote the impervious surface and the low vegetation classes, respectively. Note that evaluations for each class are presented in the form of IoU (%), and optimal values are presented in bold font.

**Fig 5:**
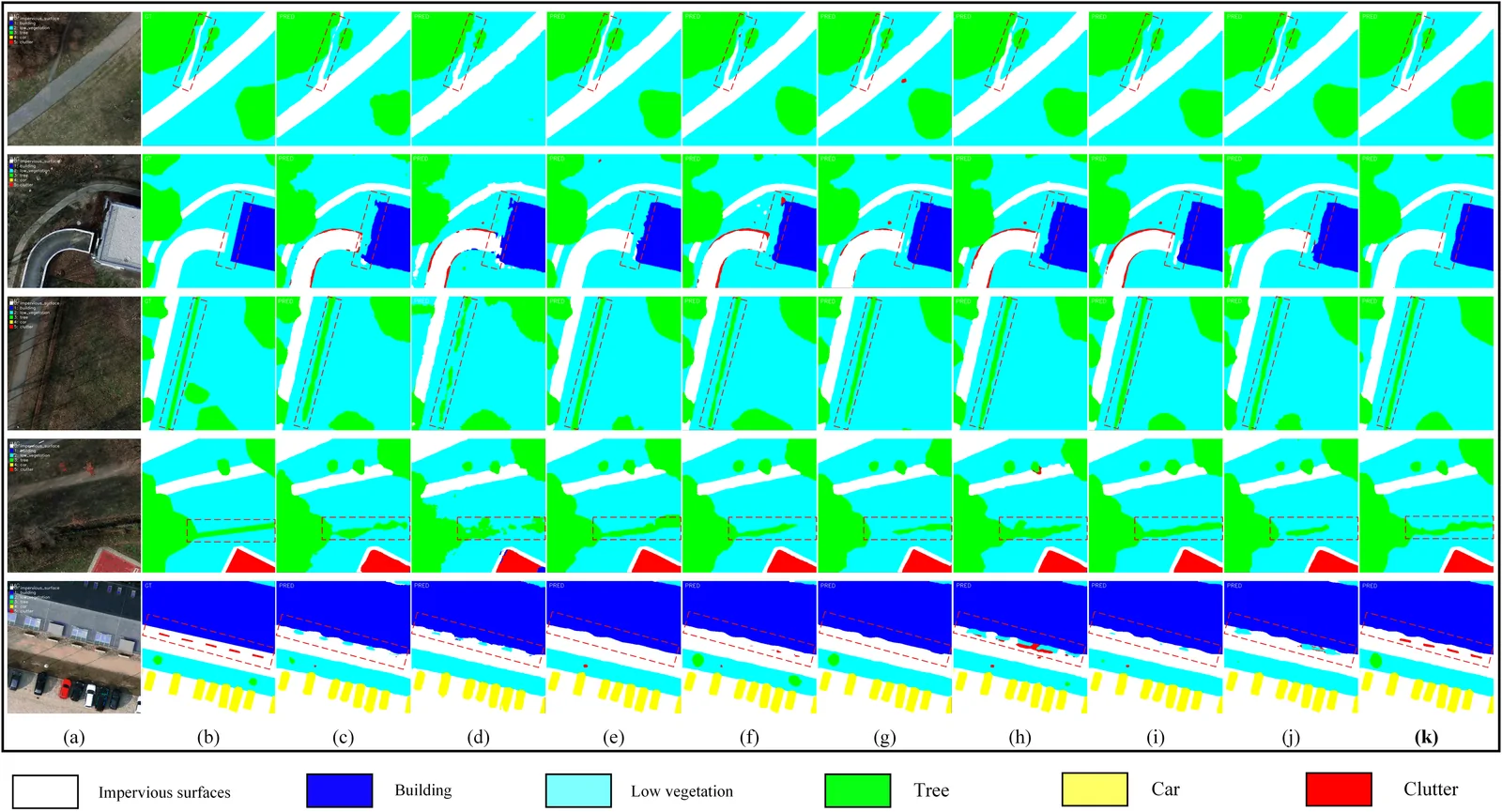
Qualitative comparison results of different methods on the Potsdam dataset. (a) original, (b) GT, (c) DeepLabV3 + , (d) SegFormer, (e) Swin Transformer, (f) PointRend, (g) CCNet, (h) UNetFormer, (i) LoG-CAN, (j) Baseline, (k) Ours.

Fig 5 shows qualitative comparison results on the ISPRS Potsdam dataset. In the regions marked by red dashed boxes, DeepLabV3 + , SegFormer, and Swin Transformer tend to produce boundary drift at the intersections of roads and buildings, which may also cause missing building edges or inconsistent road widths. PointRend and UNetFormer often produce breaks or adhesion errors in thin structures, resulting in reduced structural continuity. LoG-CAN tends to generate scattered misclassifications in regions with complex textures. In comparison, the baseline produces relatively coherent overall contours, but local fragmentation still appears around narrow boundaries and small objects. BTSR-Net achieves better alignment with the ground truth in these regions, showing higher boundary accuracy and improved structural continuity. Boundary drift is reduced, and linear structures remain more continuous and consistent.

### Comparison on the Vaihingen dataset

Table 3 reports the comparison results of different methods on the ISPRS Vaihingen dataset. Compared with Potsdam, the Vaihingen dataset contains more regular building and road structures, but also a higher concentration of small objects and narrow structures, which makes segmentation models more prone to boundary breaks and object merging errors. On this dataset, BTSR-Net achieves strong performance across all six classes, achieving an overall mIoU of 93.94%, which is higher than the baseline value of 91.67%. From the class-wise perspective, the improvements are most significant in the Car and Clutter categories. The IoU of Car increases from 83.30% to 86.70%, showing improved class-level segmentation performance for dense small vehicles, while the IoU of Clutter reaches 95.33%. The Low-veg and Tree categories also show consistent improvements, reaching 93.33% and 94.16%, respectively. Boundary-specific and connectivity-related performance is evaluated separately by mBIoU and clDice (mean), where BTSR-Net achieves 73.90% and 95.46%, respectively. The qualitative results in Fig 6 further illustrate the corresponding improvements in object boundaries and local structural details.

**Table 3:** Comparison Results for the ISPRS Vaihingen Dataset.

Methods	Imp-surf	Building	Low-veg	Tree	Car	Clutter	mIoU	mF1	OA	mBIoU	clDice (mean)
U-Net [16]	89.22	93.91	80.82	84.25	70.10	66.37	80.78	89.04	93.06	44.66	81.98
PSPNet [31]	92.79	96.11	87.59	89.50	78.42	88.53	88.82	93.99	95.59	55.97	90.96
DeepLabV3+ [52]	92.70	96.05	87.29	89.17	78.64	87.03	88.48	93.80	95.48	55.37	90.93
SegFormer [13]	92.25	95.56	86.24	88.56	76.56	91.08	88.38	93.72	95.15	55.52	89.39
Swin-UperNet [14]	95.20	97.55	91.91	93.00	84.95	93.60	92.71	96.17	97.15	68.47	93.89
Segmenter [54]	93.14	96.28	89.25	90.89	72.85	91.42	88.97	93.98	96.01	58.31	91.27
DPT [55]	91.37	94.92	85.17	87.88	72.50	88.22	86.68	92.70	94.67	51.68	88.82
PointRend [10]	95.94	97.88	93.29	94.27	86.51	94.52	93.73	96.73	97.63	72.76	94.76
CCNet [53]	95.22	97.40	92.33	93.50	83.51	93.26	92.54	96.07	97.23	68.37	93.98
UNetFormer [15]	94.04	96.77	89.71	91.35	81.46	92.30	90.94	95.19	96.39	62.67	92.44
LoG-CAN [56]	95.36	97.59	92.29	93.42	95.64	94.57	93.14	96.41	97.28	70.21	94.34
BevaNet [57]	91.45	95.26	83.34	86.27	77.30	88.70	87.05	92.98	94.33	52.51	88.77
SeaFormer [58]	84.31	89.99	71.86	78.68	49.38	73.69	74.65	84.81	89.61	32.06	75.04
EfficientViT[59]	89.08	93.48	80.36	84.32	63.49	84.79	82.59	90.15	92.96	42.67	83.59
Baseline	94.42	97.02	90.63	91.92	83.30	92.75	91.67	95.60	96.67	64.51	93.34
**Ours**	**96.08**	**98.05**	**93.33**	**94.16**	**86.70**	**95.33**	**93.94**	**96.84**	**97.67**	**73.90**	**95.46**

For brevity, Imp-surf and Low-veg denote the impervious surface and the low vegetation classes, respectively. Note that evaluations for each class are presented in the form of IoU (%), and optimal values are presented in bold font.

**Fig 6:**
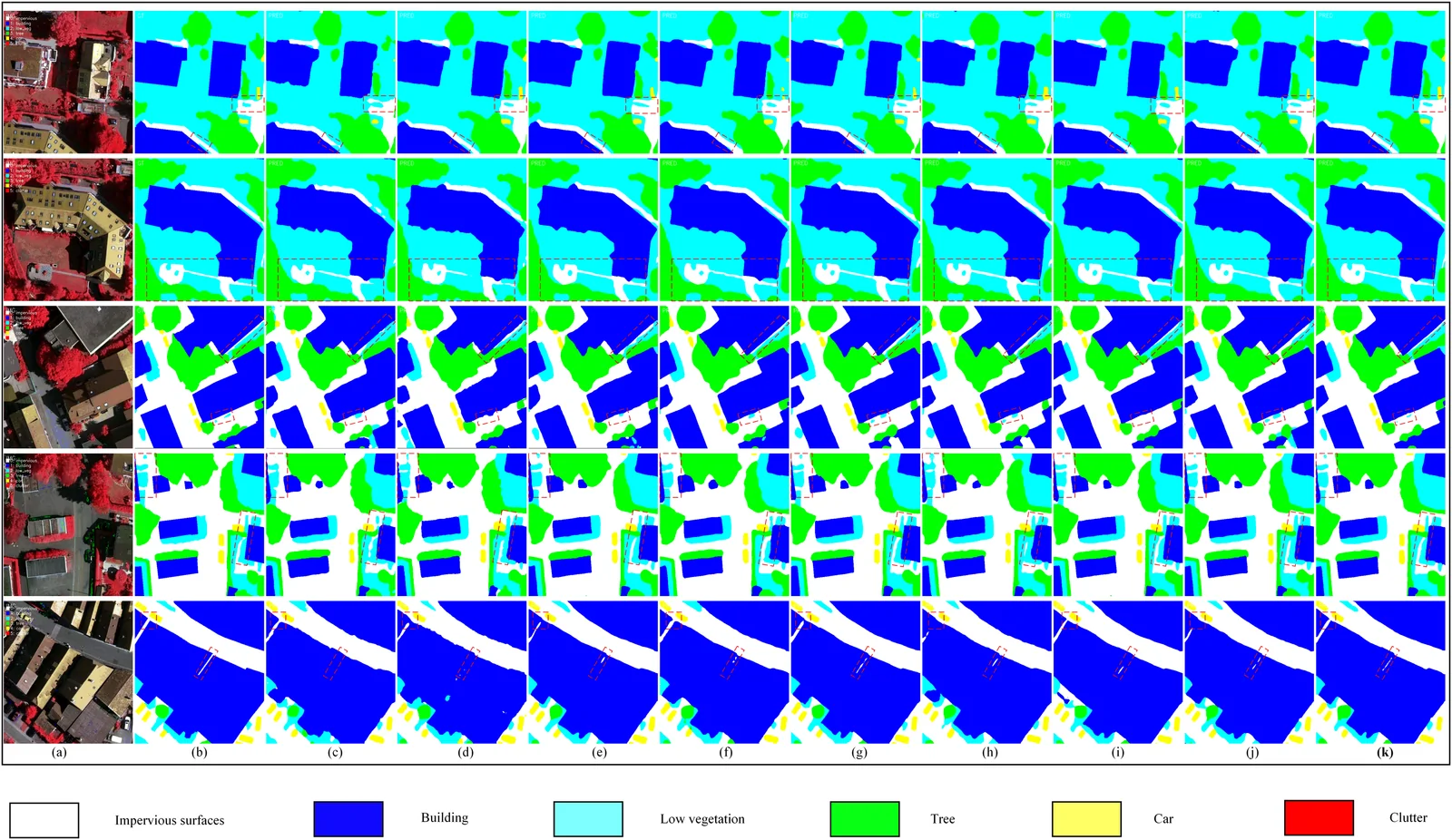
Qualitative comparison results of different methods on the Vaihingen dataset. (a) Original, (b) GT, (c) DeepLabV3 + , (d) SegFormer, (e) Swin Transformer, (f) PointRend, (g) CCNet, (h) UNetFormer, (i) LoG-CAN, (j) Baseline, (k) Ours.

Fig 6 shows qualitative comparison results on the ISPRS Vaihingen dataset. In the regions marked by red dashed boxes, DeepLabV3+ and SegFormer tend to oversmooth the boundaries between buildings and roads, causing slight expansion or shrinkage of building contours and resulting in less accurate shapes. Swin Transformer produces coherent predictions in large regions, but boundary blurring can still be observed at corners and narrow connections. PointRend and UNetFormer are more sensitive to fine details, yet they tend to generate scattered fragments in areas where tree crowns are close to buildings, resulting in noisier predictions. LoG-CAN often produces small misclassified regions near vegetation–background transitions, and adhesion errors can also be observed in some areas. The baseline produces relatively coherent contours overall, but local gaps and slight fluctuations still appear near narrow boundaries and small objects. BTSR-Net maintains clearer building boundaries and more consistent road widths in multiple highlighted regions, produces more natural transitions between trees and low vegetation, reduces small noisy fragments, and achieves higher consistency with the ground truth segmentation.

### Analysis of model complexity and inference performance

To further evaluate the model complexity and inference performance of BTSR-Net beyond accuracy improvement, this section reports the number of parameters, floating-point operations, peak GPU memory usage, single-image inference latency, and inference frame rate of each method under the same input size and hardware environment, as shown in Table 4.

**Table 4:** Comparison of Model Complexity and Inference Performance.

Methods	Params(M)	FLOPs(G)	Peak Mem(GB)	Latency (ms/img)	Inference FPS (img/s)
U-Net [16]	20.97	79.68	0.28	8.16	122.55
PSPNet [31]	48.97	356.59	0.49	15.60	64.09
DeepLabV3+ [52]	43.58	352.10	0.55	16.99	58.85
SegFormer [13]	23.07	47.39	0.39	13.12	76.20
Swin-UperNet [14]	81.15	518.83	0.74	35.40	28.25
Segmenter [54]	102.39	252.19	0.53	20.08	49.81
DPT [55]	109.67	433.32	0.67	27.71	36.08
PointRend [10]	28.70	90.50	0.29	11.64	85.89
CCNet [53]	49.82	401.73	0.45	17.87	55.96
UNetFormer [15]	11.72	23.42	0.22	7.03	142.22
LoG-CAN [56]	30.91	98.66	0.33	11.45	87.37
SeaFormer [58]	1.68	1.20	0.17	9.00	111.14
EfficientViT[59]	4.78	6.11	0.18	10.53	94.97
BevaNet [57]	56.01	54.55	0.37	17.24	58.00
Baseline	70.36	323.54	0.61	35.84	27.90
Ours	77.02	510.84	0.85	45.69	21.89

As shown in Table 4, different methods exhibit clear differences in computational complexity and inference efficiency. Lightweight models such as SeaFormer, EfficientViT, and UnetFormer have relatively low numbers of parameters and FLOPs, among which UnetFormer achieves an inference latency of 7.03 ms/img and an FPS of 142.22 img/s, demonstrating a clear speed advantage. PSPNet, DeepLabV3 + , CCNet, Swin-UperNet, and DPT have higher FLOPs and relatively increased inference latency, indicating that complex contextual modeling or Transformer-based structures introduce substantial computational overhead. BTSR-Net has 77.02 M parameters, 510.84 G FLOPs, a peak GPU memory usage of 0.85 GB, an inference latency of 45.69 ms/img, and an FPS of 21.89 img/s. Compared with the Baseline, the number of parameters of BTSR-Net increases from 70.36 M to 77.02 M, with the main overhead arising from the Geometric Boundary–Distance Branch (GBD), the Multi-scale Topology-Guided Structural Fusion (MTSF) module, and the dual-stream structural refinement module; its FLOPs increase from 323.54 G to 510.84 G, while the inference speed decreases from 27.90 img/s to 21.89 img/s. Overall, the proposed structural modules introduce additional computational cost relative to the baseline.

## Ablation study

### Overall ablation on potsdam and Vaihingen

#### Potsdam: Overall Ablation and Class-wise Gain Analysis.

Table 5 presents the ablation results on the Potsdam dataset. When HRNet+OCRNet is used as the baseline, the baseline already performs well on large-area categories, but blurred boundaries and local fragmentation still occur in regions with fine structures and complex textures. After introducing the boundary branch into the feature extraction stage, the model becomes more sensitive to contours at road–building boundaries, producing predictions closer to the real edges and improving mBIoU by 1.28%. The topology guidance with TSR fusion effectively reconnects broken structures, which is more favorable for thin structures and connected regions, leading to a 0.43% improvement in clDice (mean). Finally, after introducing the dual-stream structural refinement module, the model integrates all branches and achieves the best segmentation performance. The mIoU, mF1, OA, mBIoU, and clDice (mean) improve by 2.91%, 1.84%, 1.41%, 5.66%, and 3.74%, respectively, compared with the baseline. Overall, the modules contribute in complementary ways: the boundary branch improves boundary accuracy, Topo+TSR enhances structural connectivity, and the dual-stream refinement module combines both cues for correction within the DSR refinement narrow-band.

**Table 5:** Ablation experiments on the Potsdam dataset.

Methods	A	B	C	Imp-surf	Building	Low-veg	Tree	Car	Clutter	mIoU	mF1	OA	mBIoU	clDice (mean)
Baseline				87.60	94.70	79.22	76.86	85.04	67.32	81.79	89.73	91.56	37.51	77.89
Net1	✓			87.88	94.84	79.47	77.29	85.33	67.66	82.16	89.95	91.73	38.79	77.94
Net2		✓		87.81	94.79	79.28	77.05	85.07	67.54	81.92	89.81	91.64	37.79	77.89
Net3	✓	✓		88.10	94.79	79.43	77.16	85.56	68.37	82.24	90.01	91.75	38.83	78.32
**Ours**	✓	✓	✓	**89.72**	**95.58**	**82.38**	**79.37**	**86.24**	**74.93**	**84.70**	**91.57**	**92.97**	**43.17**	**81.63**

“A”, “B”, and “C” denote Boundary, Topo+TSR, and Struct-dual, respectively. Note that evaluations for each class are presented in the form of IoU (%).

Fig 7 shows the IoU distribution of different module combinations on the six semantic classes of the Potsdam dataset. The baseline already achieves high performance on Building and Imp-surf, and the improvements on these two classes remain limited after adding the boundary branch or topology guidance. After adding the boundary branch, the IoU of Tree and Car increases, reflecting improved class-level segmentation performance for Tree and Car. After introducing Topo+TSR, further gains are observed for Low-veg and Tree. When the boundary branch is combined with Topo+TSR, the IoU across all categories continues to improve, with the improvement being particularly noticeable for Clutter. After adding the dual-stream refinement module, Clutter shows a further increase, while Low-veg, Tree, and Car also improve. The effects on boundary quality and structural connectivity are evaluated separately by mBIoU and clDice (mean) in Table 5 and are further illustrated by the qualitative results in Figs 11 and 12.

**Fig 7:**
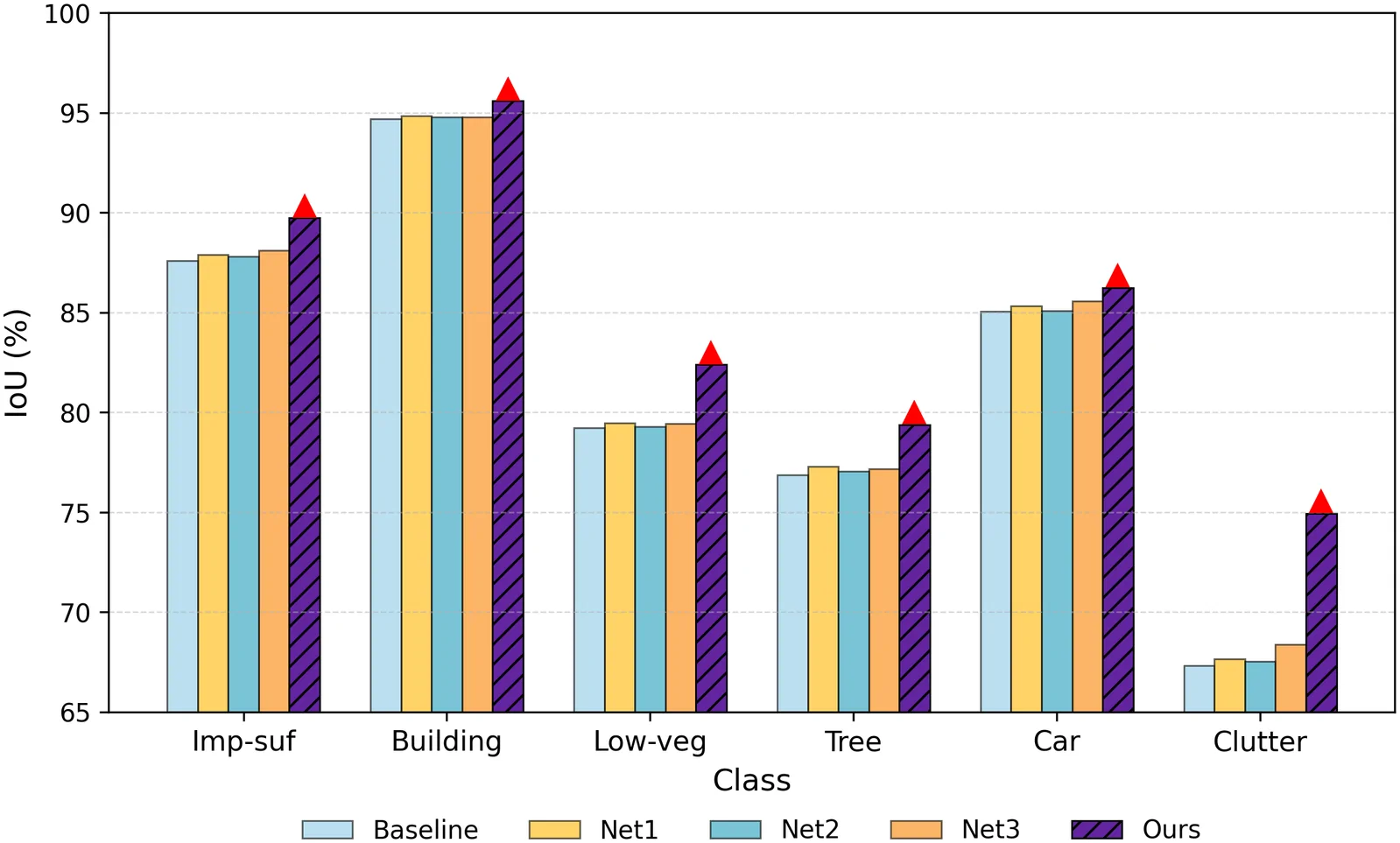
Bar Chart of Per-Class IoU for Ablation Models on Potsdam Dataset.

### Vaihingen: Overall ablation and class-wise gain analysis

Table 6 shows the ablation results on the Vaihingen dataset. After introducing the boundary branch, the IoU of Car increases from 83.30% to 85.06%, while mBIoU increases from 64.51% to 67.87%, reflecting improvements in class-level segmentation and boundary-related performance, respectively. The Topo+TSR configuration increases clDice (mean) from 93.34% to 93.83%, supporting its contribution to structural connectivity. After introducing the dual-stream refinement module, the full model achieves the largest overall gains, with mIoU, mF1, OA, mBIoU, and clDice (mean) improving by 2.27, 1.24, 1.00, 9.39, and 2.12 percentage points, respectively, compared with the baseline. Its effects on small-object boundaries and local fragmentation are further examined qualitatively in Figs 13 and 14.

**Table 6:** Ablation experiments on the Vaihingen dataset.

Methods	A	B	C	Imp-surf	Building	Low-veg	Tree	Car	Clutter	mIoU	mF1	OA	mBIoU	clDice (mean)
Baseline				94.42	97.02	90.63	91.92	83.30	92.75	91.67	95.60	96.67	64.51	93.34
Net1	✓			95.11	97.49	91.68	92.80	85.06	93.72	92.64	96.14	97.08	67.87	93.49
Net2		✓		94.46	97.04	90.72	91.99	83.37	92.39	91.66	95.60	96.69	64.82	93.68
Net3	✓	✓		95.11	97.49	91.76	92.91	84.92	93.44	92.61	96.12	97.10	68.11	93.83
Ours	✓	✓	✓	96.08	98.05	93.33	94.16	86.70	95.33	93.94	96.84	97.67	73.90	95.46

“A”, “B”, and “C” denote Boundary, Topo+TSR, and Struct-dual, respectively. Note that evaluations for each class are presented in the form of IoU (%).

Fig 8 shows the class-wise IoU results of different module combinations on the Vaihingen dataset. Building remains at a high level with little variation among models, while the differences are mainly concentrated on Low-veg, Tree, Car, and Clutter. From the baseline to the boundary branch, topology guidance with TSR fusion, and finally the dual-stream refinement module, the IoU of Low-veg and Tree increases progressively. The Car category exhibits one of the most pronounced gains, and Clutter also shows a clear improvement after introducing the dual-stream refinement branch. The change in Imp-surf is relatively small across the evaluated module configurations. The corresponding effects on boundary quality and structural connectivity are evaluated separately by mBIoU and clDice (mean) in Table 6 and are further examined qualitatively in Figs 13 and 14.

**Fig 8:**
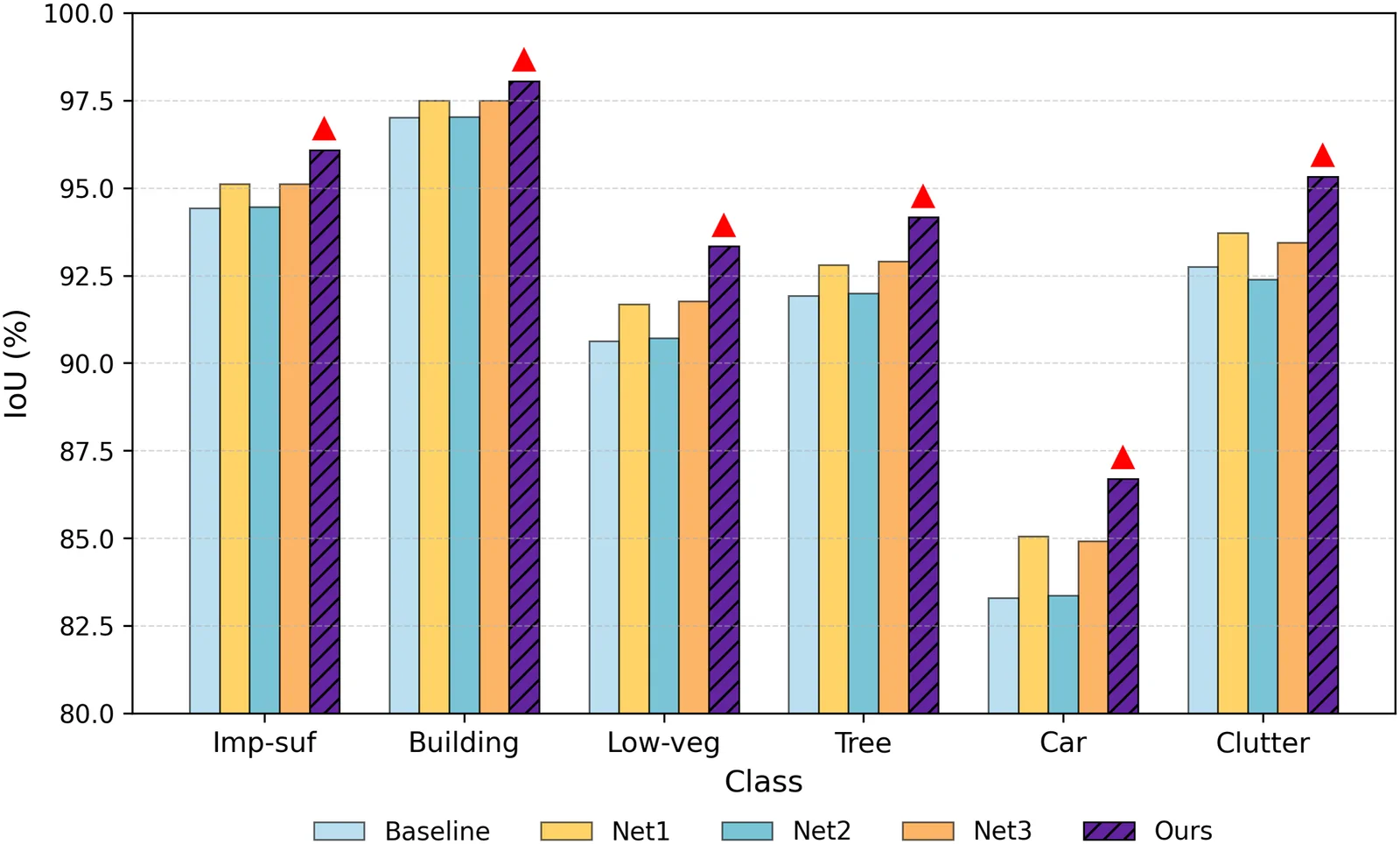
Bar Chart of Per-Class IoU for Ablation Models on Vaihingen Dataset.

### Module and hyperparameter ablation

#### Geometric boundary–distance branch.

Table 7 reports the ablation results of the GBD module on the two datasets under different supervision combinations and boundary-width settings. The module is evaluated under three configurations: using only boundary supervision (GBD-Boundary), using only distance supervision (GBD-Distance), and using both simultaneously (GBD-Full). Based on the joint supervision setting, additional experiments are conducted with different boundary widths, where the width is set to 1, 2, and 3 (GBD-Narrow / GBD-Full / GBD-Wide), to analyze the effect of supervision range.

**Table 7:** GBD Ablation Experiments on Potsdam and Vaihingen Datasets.

(a). GBD Ablation Experiments on the Potsdam Dataset								
Methods	boundary	distance	boundary_width	mIoU	OA	mF1	mBFScore	mBIoU
GBD-Boundary	✓		2	82.19	91.75	89.98	57.90	38.53
GBD-Distance		✓	–	81.84	91.61	89.75	57.51	38.27
GBD-Full	✓	✓	2	**82.41**	**91.87**	**90.11**	**58.31**	**38.90**
GBD-Narrow	✓	✓	1	82.26	91.80	90.02	58.22	38.83
GBD-Wide	✓	✓	3	82.26	91.76	90.03	58.00	38.71
(b). GBD Ablation Experiments on the Vaihingen Dataset								
Methods	boundary	distance	boundary_width	mIoU	OA	mF1	mBFScore	mBIoU
GBD-Boundary	✓		2	92.17	96.89	95.88	89.33	66.42
GBD-Distance		✓	–	92.40	97.01	96.00	89.90	67.35
GBD-Full	✓	✓	2	**92.62**	**97.12**	**96.13**	**90.27**	**68.13**
GBD-Narrow	✓	✓	1	92.61	97.10	96.12	90.11	68.11
GBD-Wide	✓	✓	3	92.56	97.10	96.09	89.96	68.03

The results show that GBD exhibits consistent improvements on both datasets. Joint boundary and distance supervision improves both boundary accuracy and overall segmentation performance, and boundary width = 2 provides the most balanced results. On the Potsdam dataset, GBD-Full outperforms using only boundary or distance supervision across mIoU, mF1, OA, and boundary-related metrics, with mBFScore reaching 58.31% and mBIoU reaching 38.90%. On the Vaihingen dataset, GBD-Full also achieves the best performance, with mIoU and mBFScore reaching 92.62% and 90.27%, respectively, and mBIoU reaching 68.13%. Regarding boundary width settings, width = 2 provides the best overall balance on both datasets, while width = 1 leads to slight performance degradation due to insufficient coverage, and width = 3 also reduces performance because non-boundary regions are increasingly included in supervision, weakening the boundary-focused constraint.

### Multi-scale topology-guided structural fusion

"Table 8a and b report the ablation settings of MTSF on the two datasets. The experiments are designed around three factors: whether to enable the topology branch (Topology Head), whether to introduce topology loss (Topo Loss, where λ=0.2 is fixed in this work), and whether to perform multi-scale fusion along with the selection of fusion levels (Fusion Scales). Based on these factors, five configurations are designed: without MTSF (w/o MTSF), using only the topology branch with topology loss but without multi-scale fusion (Topology-only), using only multi-scale fusion without topology loss (Fusion-only), enabling both topology branch and multi-scale fusion (MTSF-Full, fusion scales = All), and performing fusion only at the last stage (MTSF-FuseLast).

**Table 8:** MTSF ablation experiments on Potsdam and Vaihingen datasets.

(a). MTSF ablation experiments on the Potsdam dataset								
Methods	Topology Head	Topo Loss (λ)	Multi-scale Fusion	Fusion Scales	mIoU	OA	mF1	clDice (mean)
w/o MTSF	–	–	–	–	82.16	91.73	89.95	77.94
Topology-only	✓	0.2	–	–	82.26	91.80	90.02	78.32
Fusion-only	✓	–	✓	All	82.12	91.72	89.93	78.26
MTSF-Full	✓	0.2	✓	All	**82.39**	**91.80**	**90.12**	**78.43**
MTSF-FuseLast	✓	0.2	✓	Last	81.97	91.66	89.83	78.34
**(b). MTSF ablation experiments on the Vaihingen dataset**								
Methods	Topology Head	Topo Loss (λ)	Multi-scale Fusion	Fusion Scales	mIoU	OA	mF1	clDice (mean)
w/o MTSF	–	–	–	–	92.54	97.08	96.09	93.63
Topology-only	✓	0.2	–	–	92.57	97.08	96.10	93.68
Fusion-only	✓	–	✓	All	92.58	97.09	96.10	93.64
MTSF-Full	✓	0.2	✓	All	**92.61**	**97.10**	**96.12**	**93.68**
MTSF-FuseLast	✓	0.2	✓	Last	92.58	97.09	96.10	93.64

The ablation results show that topology supervision and multi-scale fusion provide complementary effects. On Potsdam, Topology-only increases clDice (mean) from 77.94% to 78.32%, whereas Fusion-only provides smaller gains. Combining both components in MTSF-Full yields the best results, with 82.39% mIoU and 78.43% clDice (mean). Restricting fusion to the last stage reduces mIoU to 81.97%. The same overall trend is observed on Vaihingen, where MTSF-Full achieves 92.61% mIoU and 93.68% clDice (mean), outperforming the Fusion-only and MTSF-FuseLast configurations.

### Dual-stream structural refinement

Table 9a and b report the ablation settings of DSR on the two datasets. The experiments mainly investigate the influence of two types of hyperparameters: the training weight of the refinement branch (Refine Loss) and the fusion coefficient λ during inference. Four configurations are designed: without the refinement branch (w/o DSR), using a smaller refinement supervision weight with λ fixed at 0.2 (DSR-Light), increasing the refinement supervision weight and setting λ to 0.3 (DSR-Strong), and using the best-performing combination across the evaluated settings (DSR-Full, Refine Loss = 0.4, λ=0.2).

**Table 9:** DSR ablation experiments on Potsdam and Vaihingen datasets.

(a). DSR ablation experiments on the Potsdam dataset								
Methods	Refine Loss	λ (Inference)	mIoU	OA	mF1	mBFScore	mBIoU	clDice (mean)
w/o DSR	–	–	82.26	91.80	90.02	58.22	38.83	78.32
DSR-Light	0.3	0.2	82.03	91.64	89.88	58.09	38.72	78.17
DSR-Strong	0.4	0.3	81.92	91.64	89.80	58.52	39.08	78.14
DSR-Full	0.4	0.2	**84.70**	**92.97**	**91.57**	**63.68**	**43.17**	**81.63**
**(b). DSR ablation experiments on the Vaihingen dataset**								
Methods	Refine Loss	λ (Inference)	mIoU	OA	mF1	mBFScore	mBIoU	clDice (mean)
w/o DSR	–	–	92.61	97.10	96.12	90.11	68.11	93.68
DSR-Light	0.3	0.2	92.61	97.12	96.12	90.25	68.25	93.82
DSR-Strong	0.4	0.3	92.67	97.13	96.15	90.07	68.17	93.70
DSR-Full	0.4	0.2	**93.94**	**97.67**	**96.84**	**94.21**	**73.90**	**95.46**

On the Potsdam dataset, DSR-Full increases mBFScore and mBIoU from 58.22% and 38.83% to 63.68% and 43.17%, respectively, while clDice (mean) increases from 78.32% to 81.63%. On Vaihingen, DSR-Full improves mBFScore, mBIoU, and clDice (mean) by 4.10, 5.79, and 1.78 percentage points, respectively. Increasing the inference fusion coefficient to 0.3 in DSR-Strong does not provide further improvement: the Potsdam mIoU decreases to 81.92%, and the Vaihingen clDice (mean) is also lower than that of DSR-Full. Therefore, the selected fusion setting retains the primary prediction while using the refinement output as a supplementary correction for structural errors.

### Boundary–topology consistency weight

This section evaluates the sensitivity of the boundary–topology consistency weight $w_{BT}$ defined in the overall training objective. With all other settings of the full model fixed, $w_{BT}$ is varied among 0.00, 0.02, and 0.05. The corresponding changes in mIoU, OA, mF1, mBFScore, mBIoU, and clDice are evaluated on both datasets, as shown in Figs 9 and 10.

**Fig 9:**
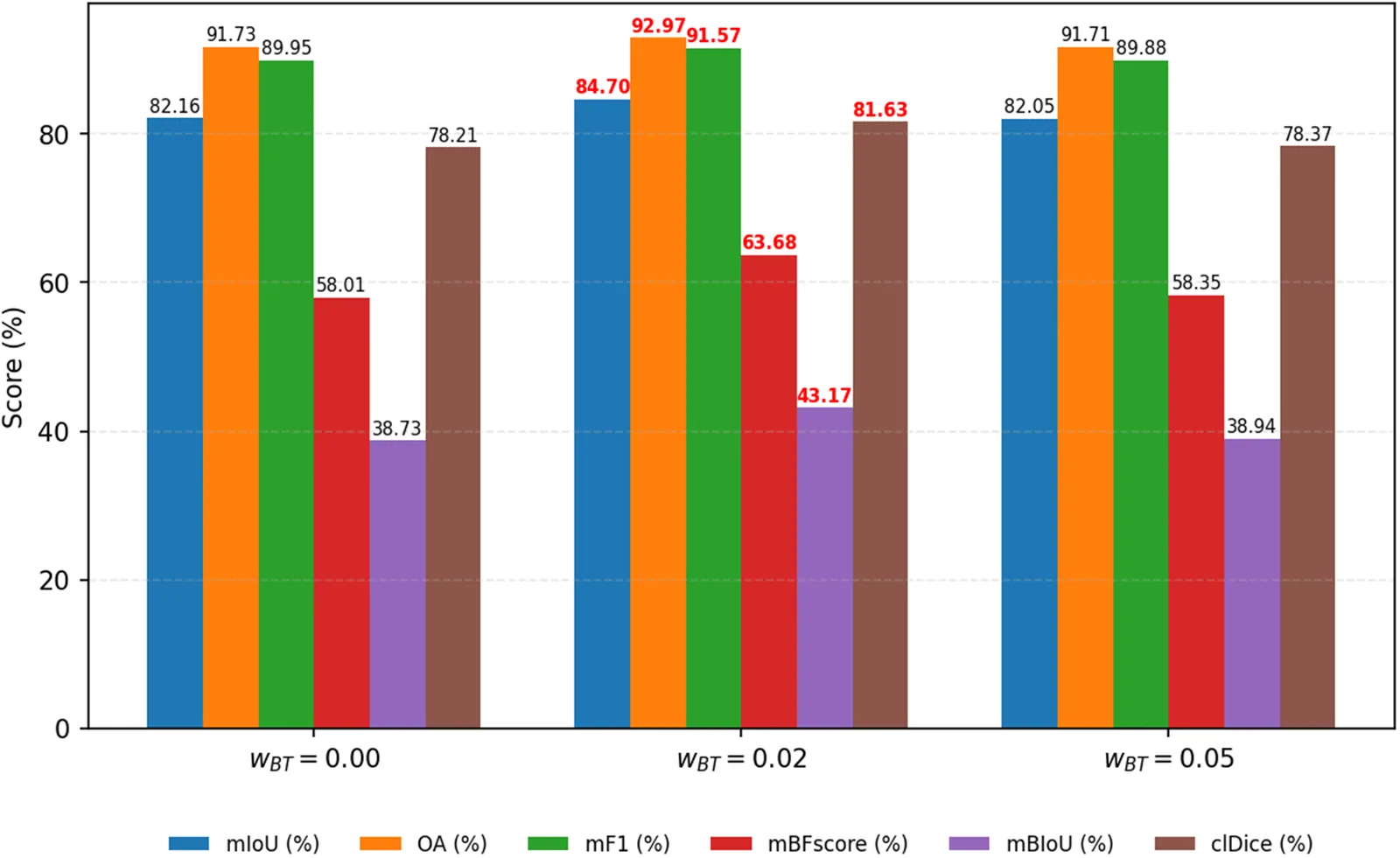
Ablation results for boundary–topological consistency weights on the Potsdam dataset.

**Fig 10:**
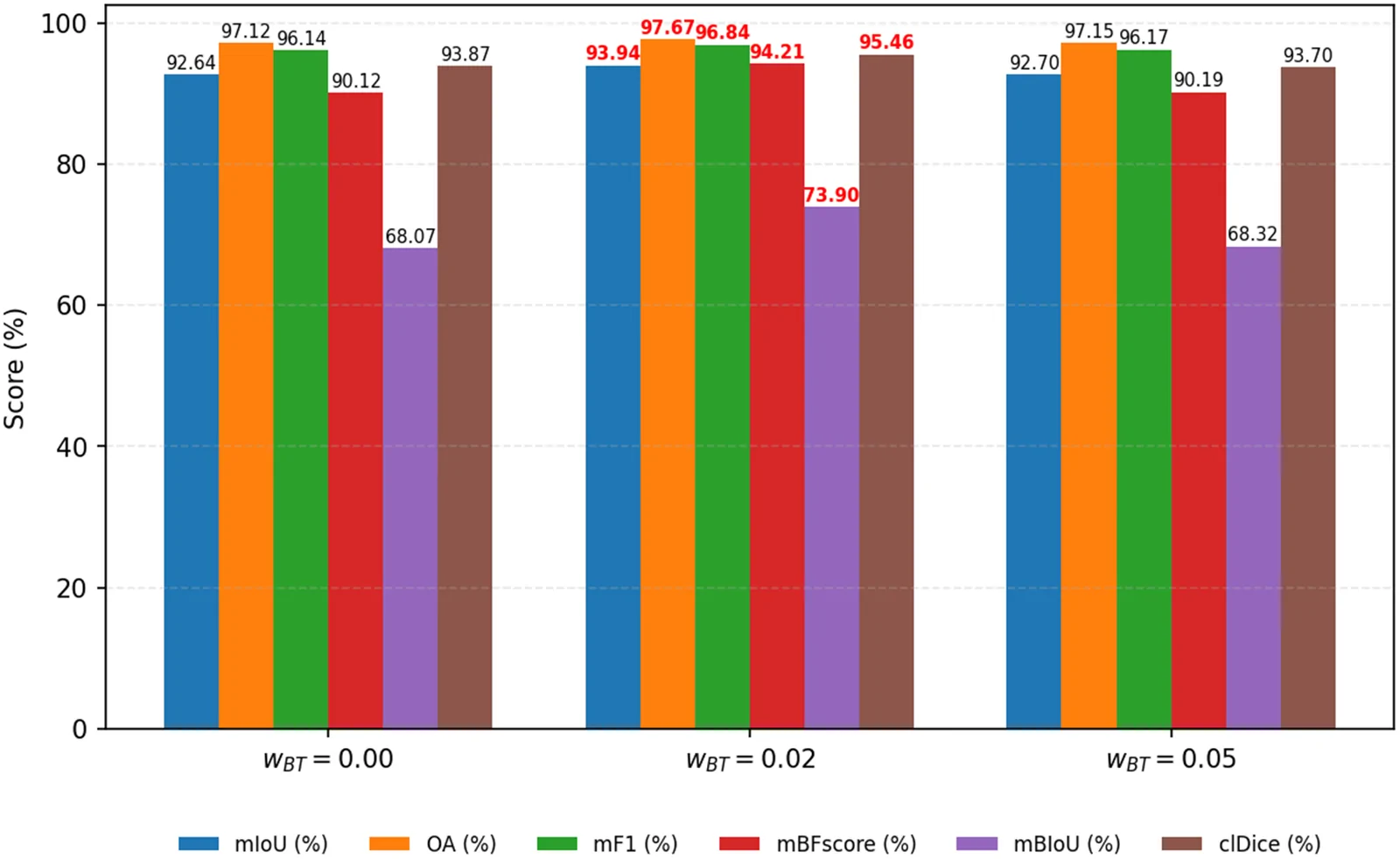
Ablation results for boundary–topological consistency weights on the Vaihingen dataset.

As shown in Figs 9 and 10, compared with $w_{BT}=0.00$, setting $w_{BT}=0.02$ provides the best overall balance on both datasets, with clearer improvements in mBFScore, mBIoU, and clDice. When $w_{BT}$ is further increased to 0.05, both overall and structural metrics decline, suggesting that excessive consistency regularization may suppress region discrimination.

### Visualization-based analysis of segmentation and structure

This section analyzes the segmentation quality and structural performance of the proposed model through qualitative visualization results. Two types of visualizations are presented: RGB segmentation results are used to observe region completeness and boundary accuracy, and binary structure maps are used to examine the continuity of thin boundaries and linear structures, with a focus on breaks, adhesion errors, and local fragmentation. Dashed boxes are used to mark error-prone regions for direct comparison of different methods at the same locations. The comparison mainly focuses on the boundary between Building and Imp-surf, the transition region between Tree and Low-veg, and areas with dense small objects such as Car and Clutter.

### Visualization comparison on the potsdam dataset

To facilitate comparison among different ablation settings, Fig 11 is organized in the order of “original image–GT–predictions from each model”, where the prediction results correspond to Baseline, Net1, Net2, Net3, and Ours.

**Fig 11:**
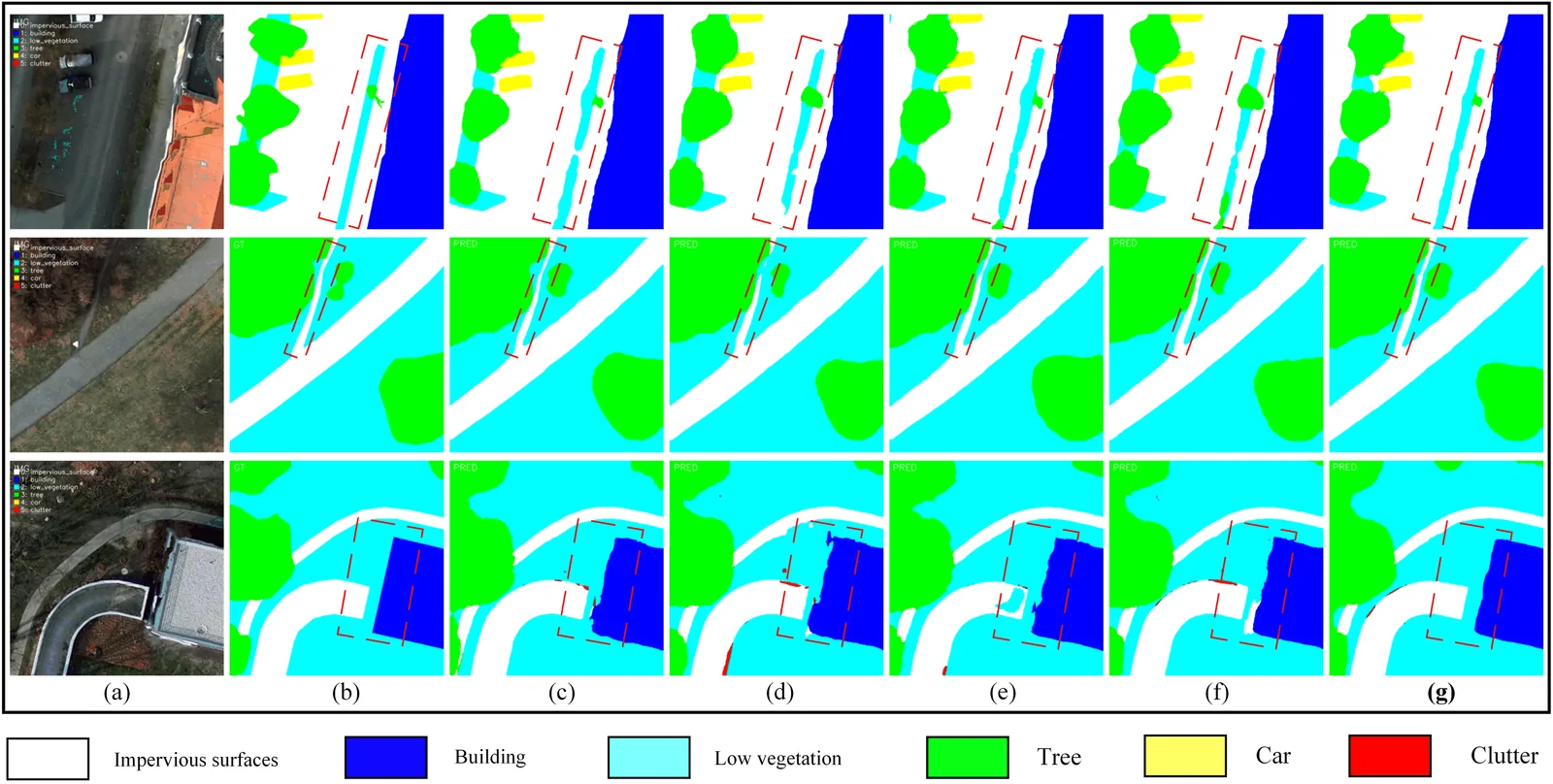
Visualization results of RGB semantic segmentation from the Potsdam dataset using the ablation model.

Fig 11 shows RGB visualization results of different ablation configurations on the Potsdam dataset. The red dashed boxes highlight two types of error-prone regions: the boundary regions between roads and low vegetation/trees, and the narrow structures at the intersection of building edges and roads. The baseline separates the main structures in these regions, but boundary drift frequently occurS, narrow regions become thicker or broken, and low vegetation near road edges may be incorrectly merged into impervious surfaces. After Net1 incorporates Boundary, the contour lines align more closely with the ground truth, and the jaggedness along the building edges is reduced; however, discontinuities still appear in narrow areas, indicating that boundary cues alone are not sufficiently reliable. After introducing Topo+TSR in Net2, thin structures in the boxed regions become more continuous, with fewer breaks, although slight adhesion errors may still occur. When both Boundary and Topo+TSR are used in Net3, boundary positions and connectivity become more consistent, narrow regions at building–road intersections are cleaner, and misclassification near boundaries is reduced. After further introducing Struct-dual in our model, the narrow structures in the boxed regions are closer to the GT in both thickness and orientation, boundaries around small objects are more complete, and local breaks and adhesions are reduced. To further analyze differences in boundary accuracy and structural connectivity, Fig 12 shows the GT boundaries and predicted boundaries of each ablation model side by side, with red boxes marking narrow regions and small-object areas that are prone to errors.

**Fig 12:**
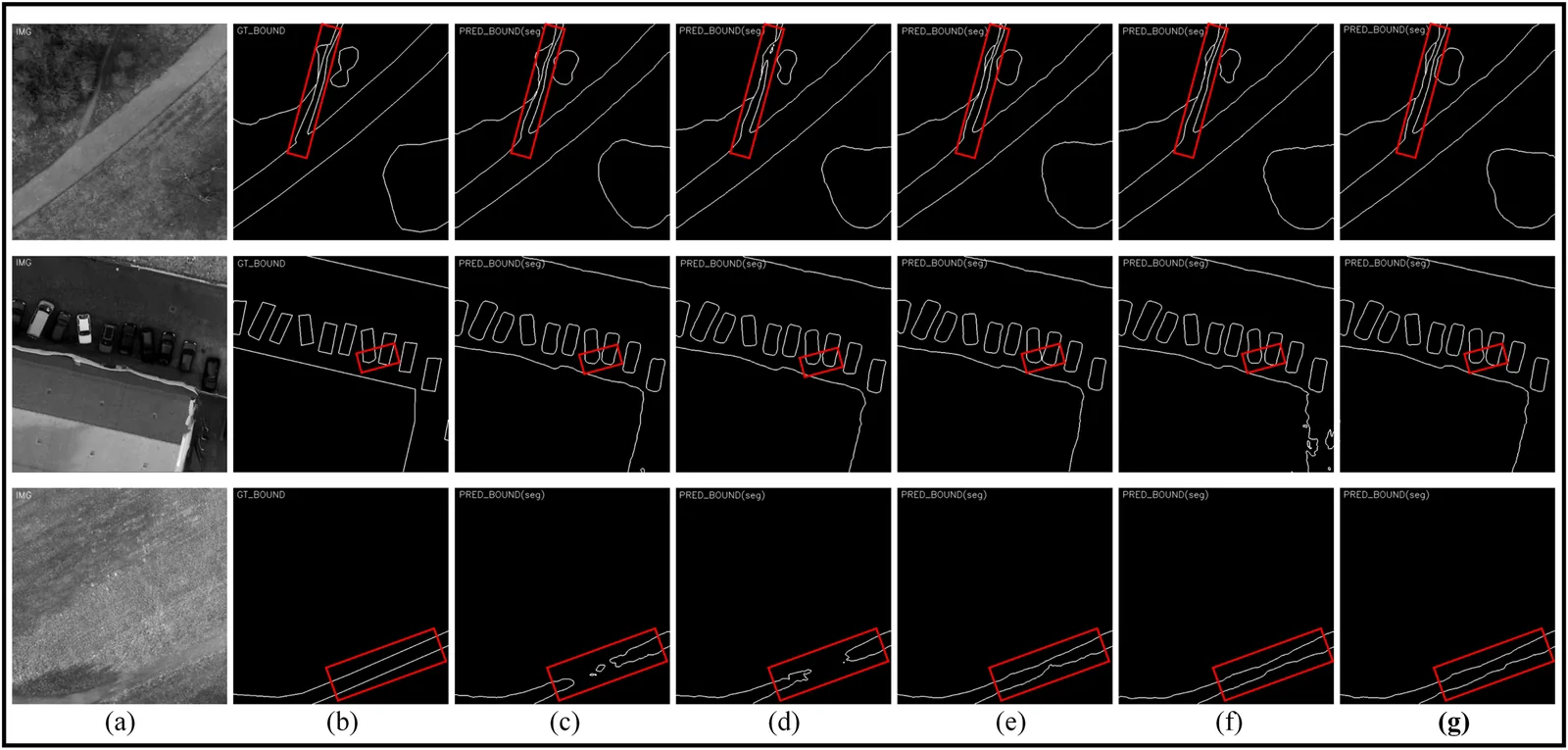
Visualization of boundary structures in the Potsdam dataset.

The red boxes in Fig 12 mainly cover three typical cases: thin road lines, dense small objects (such as vehicle contours), and narrow structures on weak-texture backgrounds. The baseline prediction shows two common problems: broken or thickened lines causing discontinuous thin structures, and incomplete boundaries of small objects that become flattened into short segments. After adding the boundary branch, boundary locations near road lines become closer to the GT, line thickness becomes more consistent, and small object contours are easier to preserve. After introducing topology guidance and TSR constraints, breaks are further reduced, thin structures remain more continuous, and boundary continuity at corners improves. After adding the dual-stream refinement module, fewer artifacts and gaps appear in the boxed regions, small object contours become more complete, and overall boundaries are closer to the GT in both shape and structural connectivity.

### Visualization comparison on the Vaihingen dataset

To further analyze the influence of each module on boundary accuracy and structural connectivity in the Vaihingen dataset, Fig 13 and Fig 14 present visualization results of the Baseline, models with Boundary and Topo+TSR added step by step, and the full model in typical regions.

**Fig 13:**
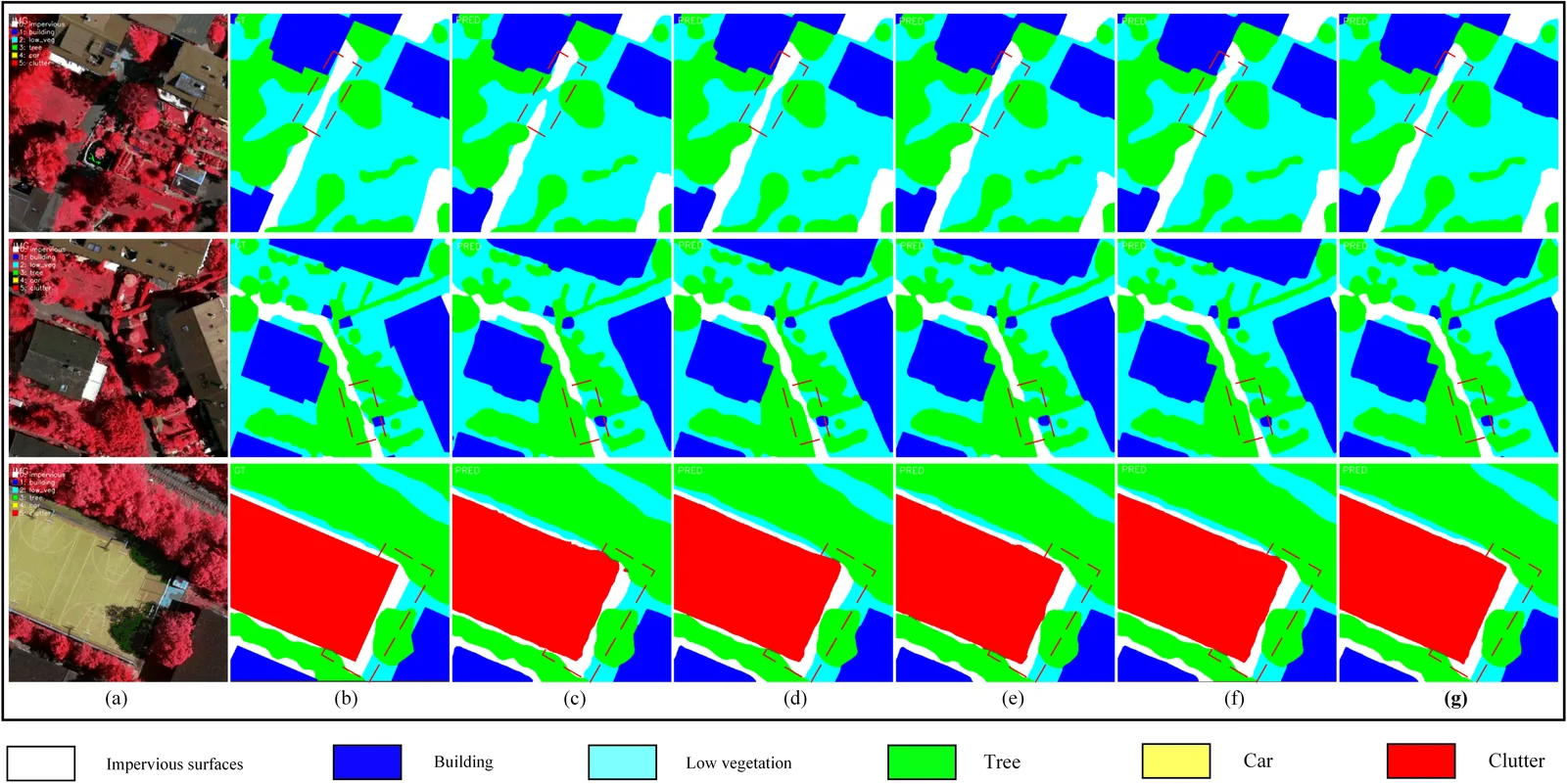
Visualization results of RGB semantic segmentation from the Vaihingen dataset using the ablation model.

**Fig 14:**
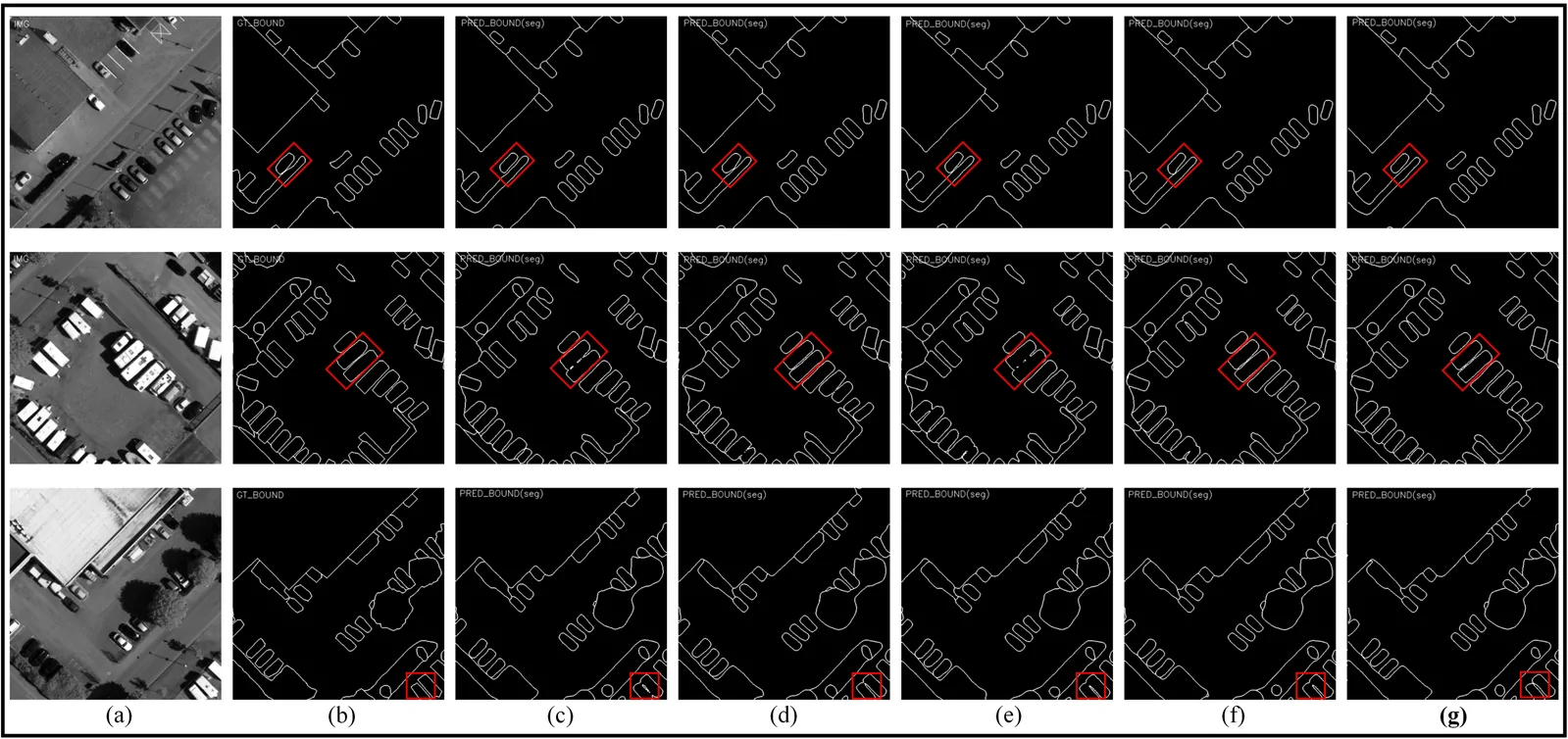
Visualization of boundary structures in the Vaihingen dataset.

Fig 13 shows segmentation results under different ablation settings on the Vaihingen dataset. In the transition regions marked by red dashed boxes, the baseline prediction tends to show boundary drift between roads and impervious surfaces and vegetation, where narrow regions may become flattened or show inconsistent widths. After adding the Boundary branch, boundary locations become closer to the GT, and jagged edges at building corners and road boundaries are reduced. After introducing Topo+TSR, thin structures show fewer breaks, and the boundary between vegetation and roads becomes more continuous. When Boundary and Topo+TSR are used together, narrow structures and small objects in the boxed regions are less likely to be merged into neighboring large regions. The complete model exhibits more complete contour closure and clearer class boundaries in the aforementioned regions, with relatively fewer misclassified fragments.

Fig 14 further presents boundary structure maps to show differences in contour continuity among models. The baseline tends to produce breaks, missing segments, or adhesion errors in regions with dense small objects or fragmented boundaries, such as vehicle contours near parking areas and road edges, resulting in incomplete contours. After adding the Boundary branch, the main contours become clearer, but discontinuities may still remain in long edges and thin structures. With Topo+TSR, the model becomes more sensitive to structural breaks, and the boundaries in the boxed regions appear more continuous with fewer gaps; when both modules work together in the full model, boundary curves become closer to the GT connectivity pattern, fragmented contours become more complete, and spurious edges are more effectively suppressed.

## Conclusion

Experimental results on the ISPRS Potsdam and Vaihingen datasets demonstrate that BTSR-Net improves overall segmentation accuracy while providing additional gains in boundary quality and structural connectivity. The ablation studies further confirm that boundary-aware geometric modeling, connectivity-oriented topology modeling, and localized structural refinement contribute complementary improvements to the final segmentation results. Overall, these results validate the effectiveness of jointly exploiting boundary and connectivity information for fine-grained semantic segmentation of high-resolution remote sensing imagery.

Despite these improvements, the additional structural modeling introduces non-negligible computational overhead. Compared with the baseline, the number of parameters increases from 70.36 M to 77.02 M and the FLOPs increase from 323.54 G to 510.84 G, while the inference speed decreases from 27.90 img/s to 21.89 img/s. This computational cost may limit the applicability of the current model in resource-constrained or real-time scenarios. Future work will therefore focus on improving computational efficiency through more lightweight boundary and topology modeling, reducing redundant multi-scale feature computation, and exploring more selective refinement strategies while preserving the structural modeling capability of the proposed framework.
